# A novel optically gated thin-film transistor sensor for real-time chemical differentiation using machine-learning analysis

**DOI:** 10.1016/j.biosx.2026.100784

**Published:** 2026-04-17

**Authors:** Lukas M. Crockett, Jacob Jackson, Tucker P. Gratton, Jenée D. Cyran, Bamidele Omotowa, Kristy A. Campbell

**Affiliations:** a Department of Electrical and Computer Engineering, Boise State University, Boise, ID, United States; b Department of Chemistry, Boise State University, Boise, ID, United States; c Pearlhill Technologies, Idaho Falls, ID, United States

**Keywords:** Optically gated transistor, Transient response, Pulse sequence, Machine learning, PFAS, Chemical differentiation, Ultra-short chain, Unfunctionalized sensor

## Abstract

We present an optically gated thin-film transistor sensor that distinguishes chemicals through illumination-induced transient electrical responses. The device consists of a p-type silicon substrate with native oxide and an amorphous Ge_2_Se_3_ photogating layer, and operates without reference electrodes or surface functionalization. Structured light-pulse sequences produce time-dependent electrical signatures whose features differ across chemical environments. These transient responses are analyzed using supervised machine-learning models to demonstrate real-time chemical differentiation in controlled solvent-prepared samples. As a demonstration, we classify three alcohols (methanol, ethanol, and isopropanol) and three perfluoroalkyl substances (perfluorooctane, perfluoropentanoic acid, and perfluoropropionic acid), each measured at 1 ppm in methanol. Waveform-derived features enable machine-learning classification with accuracies of 95–99% and F1 scores of 0.85–0.96, while a total organic fluorine classifier achieves 94% accuracy. These results show that a simple optically gated Si-based transistor can be used as a compact and generalizable sensor architecture for real-time chemical identification.

## 1. Introduction

Per- and polyfluoroalkyl substances (PFAS) are increasingly scrutinized across industrial, environmental, and biological settings due to their persistence and mobility (Delatour et al., 2023; Grung et al., 2024; Peng et al., 2025; Saha et al., 2025; Zhou et al., 2024). Rapid identification of PFAS following a suspected release remains challenging because available analytical methods require centralized laboratory infrastructure and extended turnaround times (Rodriguez et al., 2020; Yaghoobian et al., 2025). In many field scenarios, such as evaluating soil, surface residues, or process materials, the only practical approach to minimizing chemical release is to prepare a small field-collected sample in a simple solvent such as methanol, and perform an immediate measurement. However, this is currently not feasible due to the absence of real-time sensing tools for such field measurements, limiting rapid assessment of PFAS presence directly at the point of sample collection (Bayode et al., 2024; Kobayashi et al., 2024).

A wide range of PFAS sensing technologies has been explored for real-time detection, including electrochemical platforms, molecularly selective interfaces, nanoparticle-based systems, metal-organic framework devices, molecularly imprinted sensors, photonic structures, and field-effect transistor architectures (Clark and Dick, 2021; Huo et al., 2025; Mirabediny et al., 2023; Nahar et al., 2023; Ou et al., 2025). Although these strategies have achieved important advances, many rely on specialized surface functionalization, liquid-liquid interfaces, molecular imprinting, or structured framework materials (Adaryan et al., 2025; Barua et al., 2024; Cheng et al., 2025; Lu et al., 2026; Roh et al., 2025). Such equilibrium-based or receptor-dependent approaches can introduce challenges related to stability, reproducibility, and calibration in field-prepared samples. These limitations highlight the need for sensing architectures that do not depend on selective surface chemistry, but instead extract chemical information from intrinsic device dynamics. Related efforts in material-driven and bioinspired sensing have similarly emphasized the use of intrinsic material properties and engineered interfaces for signal generation in diagnostic and analytical applications (Mahato et al., 2018; Marimuthu et al., 2025). These approaches reinforce the broader shift toward sensing strategies that rely on dynamic material responses rather than dedicated receptor layers.

**Fig. 1.**
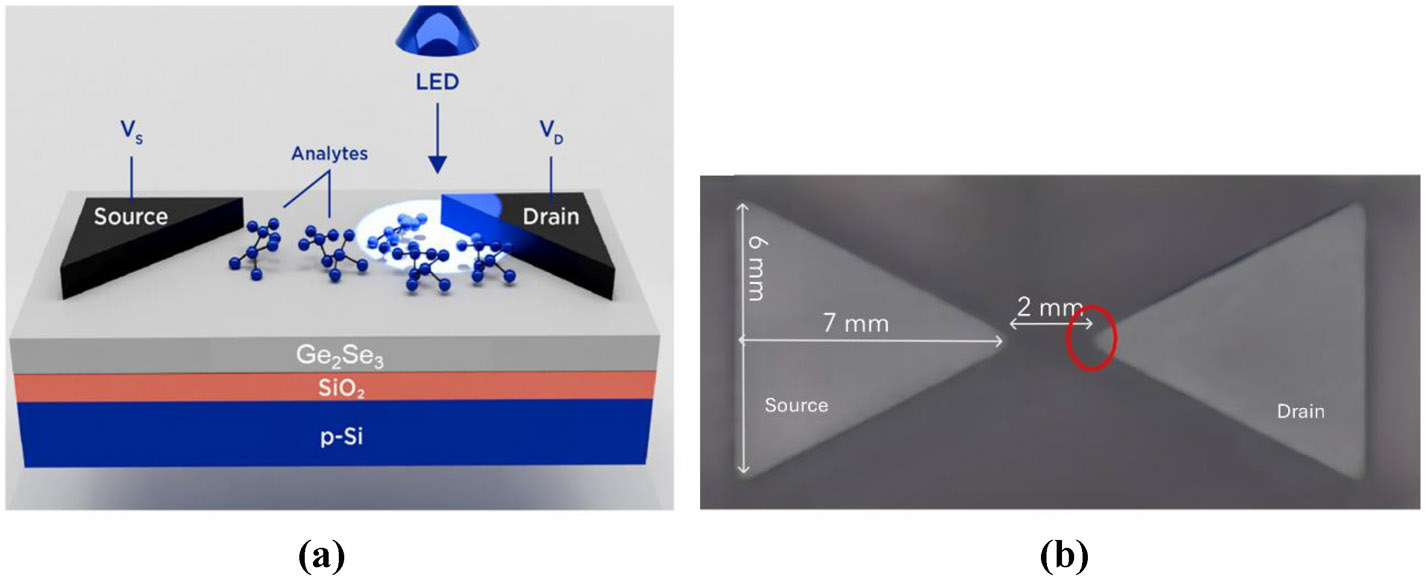
**(a)** ENVIR-OGT device structure; **(b)** top-down image with the red circled region corresponding to illumination and sample placement location.

The Environmental Optically Gated Transistor (ENVIR-OGT) investigated in this work builds on earlier optically gated transistor concepts (Kabir and Campbell, 2024) and addresses real-time chemical sensing by using illumination-driven transient perturbations to reveal analyte-dependent modifications of device interfacial electrostatics that alter electrical response. The ENVIR-OGT structure, shown in Fig. 1, is a simplified version of the original OGT and consists of a silicon substrate with native oxide, a single amorphous Ge_2_Se_3_ photogating layer, and laterally spaced source and drain metal electrodes defining the conduction pathway. The original OGT was developed as an optically gated access transistor for resistive random-access memory and consisted of three Ge_2_Se_3_ layers alternated with two M + Ge_2_Se_3_ layers, where M denotes a metal dopant such as Cu or Sn (Campbell et al., 2019). During operation, it was observed that the channel current was influenced by atmospheric conditions, prompting investigation of the device as a chemical sensor. For sensing applications, the structure was optimized to a single Ge_2_Se_3_ layer and showed enhance environmental sensitivity.

While individual light pulses produce measurable responses in the presence of analytes, a single pulse does not provide sufficient information to differentiate most chemical classes, particularly under variable concentration conditions. The detection and classification strategy therefore draws inspiration from magnetic resonance methodologies, in which structured pulse sequences probe interaction dynamics through controlled timing.

Initial studies support an operational mechanism in which illumination perturbs trap occupancy and interfacial electrostatics within the Ge_2_Se_3_ layer while the underlying silicon responds through its own photogenerated charge dynamics. These coupled photoinduced processes generate transient electrical responses that carry chemically specific information. Because the device requires no reference electrode or selective surface functionalization, its operation remains compatible with simple solvent-prepared samples and does not rely on adsorption-driven equilibrium processes.

This work evaluates the ENVIR-OGT as a general approach for chemical differentiation based on structured transient optical stimulation. The device is probed using sequences of light pulses designed to access both fast and slow components of the photoinduced response, producing time-dependent electrical waveforms whose features vary with the chemical environment. Chemical differentiation arises from how each environment perturbs the temporal evolution of the device response rather than from shifts in a static operating point. Extracted waveform features are used as inputs to supervised machine-learning classifiers.

To demonstrate capability, three common alcohols and three representative PFAS were examined at a fixed concentration in methanol as controlled solvent-prepared samples. The ENVIR-OGT generates reproducible transient signatures across analytes and devices, enabling accurate identification using both individual-class and total organic fluorine classifiers. This study emphasizes proof-of-concept chemical differentiation at a fixed concentration and does not pursue analytical performance metrics such as detection limits or matrix tolerance, which are reserved for future investigation.

## 2. Methods

### 2.1. Chemicals tested

Three neat alcohols tested were used as purchased: Methanol (MeOH) (Fisher Scientific A412-1, 99.9%); ethanol (EtOH) (Decon Labs, Inc. 200 Proof, undenatured); and isopropyl alcohol (IPA) (Fisher Scientific 99.9%).

All PFAS samples were prepared using methanol (MeOH; >99.8% Sigma 179337). The PFAS samples were prepared as follows: A stock solution of ***Perfluorooctane*** (**C_8_F_18_;** Sigma-Aldrich 98%, product number 359238) was prepared by placing 0.0566 mL of **C_8_F_18_** into 9.943 mL MeOH. 10 μL of the **C_8_F_18_** stock solution was added to 990 μL of MeOH in order to achieve hundred-fold dilution, then 10 μL of this diluted preparation was added to 990 μL of MeOH to achieve a 1 ppm solution. A stock solution of ***Perfluoropentanoic acid*** (**PFPeA**; 97%, 5 mL; Sigma 396575) was prepared by placing 0.0584 mL PFPeA into 9.942 mL MeOH. 10 μL of this stock solution was added to 990 μL of MeOH in order to achieve hundred-fold dilution. 10 μL of this diluted preparation was then added to 990 μL of MeOH to achieve a 1 ppm solution. A stock solution of ***pentafluoropropionic acid*** (**PFPrA**; 97%, Sigma 245917) was prepared by placing 0.0635 mL into 9.937 mL MeOH. 10 μL of stock solution was added to 990 μL of MeOH in order to achieve hundred-fold dilution. 10 μL of this diluted preparation was added to 990 μL of MeOH to achieve a 1 ppm solution. The dilutions were used within four days.

**Fig. 2.**
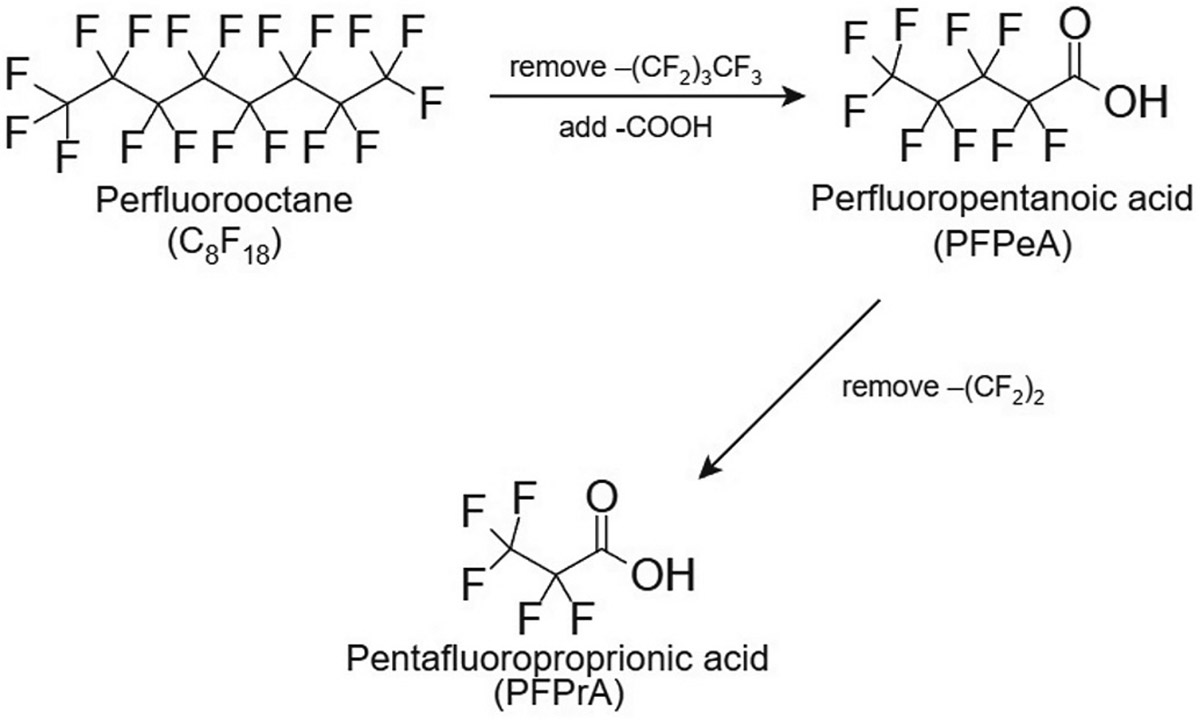
PFAS measured, emphasizing structural differences.

The PFAS were selected due to their structural progression, from the straight chain eight carbon alkyl fluoride, perfluorooctane to the ultrashort chain, **PFPrA** (Fig. 2). These different PFAS were desired in order to measure the ability of the ENVIR-OGT to sense small changes between PFAS structure and chain length, in addition to measurement of Total Organic Fluorine (TOF).

To summarize, the samples tested were:

1. Alcohols: MeOH, EtOH, and IPA, neat.
2. PFAS: C_8_F_18_, PFPeA, and PFPrA, each diluted to 1 ppm in MeOH.

### 2.2. OGT fabrication

The ENVIR-OGT is a small device, with a die size of approximately 20 mm × 10 mm (Fig. 1). Portions of the a-Ge_2_Se_3_ layer are left exposed to illumination and the test liquid. Devices were fabricated on 100 mm p-Si wafers with a native SiO_2_ layer. A 20 to 25 nm thick a-Ge_2_Se_3_ film and 35 nm thick W electrodes were deposited by sputtering. The W source–drain electrodes were spaced approximately 2 mm apart. Portions of the a-Ge_2_Se_3_ surface between the electrodes were left intentionally exposed to illumination and to the test solution. The wafer was diced into 20 × 10 mm chips and tested without encapsulation. Additional fabrication details are provided in the Supplementary Materials.

### 2.3. ENVIR-OGT electrical measurements

#### 2.3.1. Equipment and test setup

**Fig. 3.**
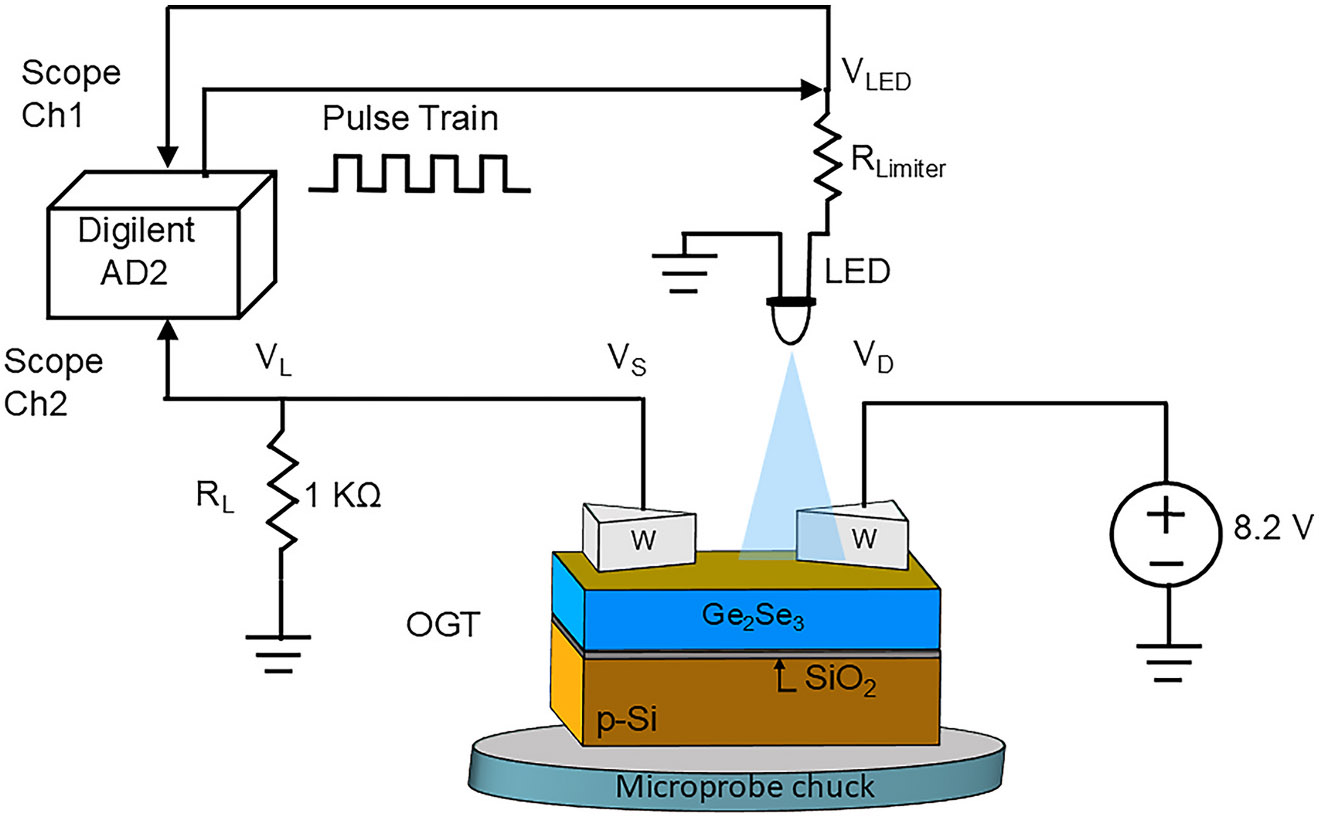
Electrical measurement platform.

**Fig. 4.**
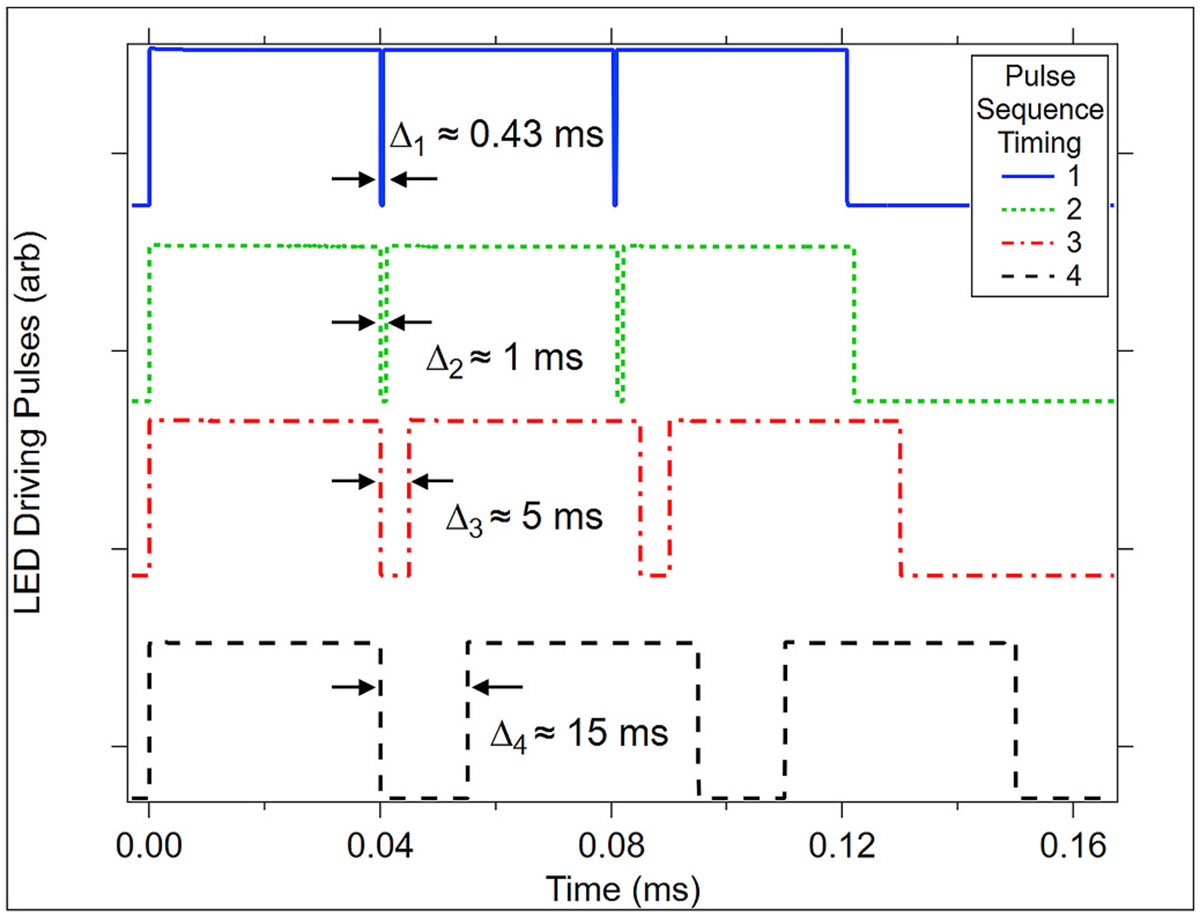
Pulse sequence timing. A 1 s wait is applied between pulse train sequences.

All chemical sample testing electrical measurements were performed using a Micromanipulator microprobe station (SM, Fig. S2). Steady-state I–V curves were obtained with a Keysight B1500 parameter analyzer, using double sweeps between −10 V and +10 V. Time-series pulse measurements were collected using the test setup shown in Fig. 3, which employed a Digilent Analog Discovery 2 (AD2) for synchronized waveform generation and data acquisition through its oscilloscope channels. The AD2 arbitrary-waveform output gated a “white-light” LED (DigiKey LTW-2S3D7) through a current-limiting resistor ($R_{\text{Limiter}}$), producing the pulse sequences shown in Fig. 4. There was a 1 s delay between each of the four pulse sequences. A 1 kΩ load resistor ($R_{L}$) was connected in series between the source electrode and ground, while an 8.2 V battery pack biased the drain ($+V_{D}$) relative to the resistor ground reference. The battery was used to minimize any electronic noise. One oscilloscope channel recorded the LED driver waveform ($V_{\text{LED}}$), and the second simultaneously monitored the voltage across $R_{L}$ ($V_{L}$, equivalent to $V_{S}$). The channel current was calculated as $I_{DS}=V_{L}∕R_{L}$. Since $V_{DS}$ varied slightly with $I_{DS}$ due to voltage division across $R_{L}$, a drain bias of 8.2 V was selected to ensure operation in the saturation region under baseline air detection conditions. The resulting variations in $V_{DS}$ (<600 mV) are minor and do not affect $I_{DS}$ interpretation in this regime; these small variations are considered intrinsic to the chemical-identification signal produced by the ENVIR-OGT. Note that that the larger the amplitude of the measured response (which is the potential difference between the source electrode, $V_{s}$, and ground, and is identical to the potential across $R_{L}$), the larger the channel current, $I_{DS}$.

The “white-light” LED can be replaced with LEDs of various wave-lengths (UV to near-IR), enabling wavelength-dependent selectivity measurements.

#### 2.3.2. Electrical measurements

Each measurement pulse sequence was initiated with an air measurement to establish a device baseline response, followed by sample analysis. After the baseline air measurement, 1 μL of the test solution was dispensed onto the ENVIR-OGT surface adjacent to the drain electrode (contact region highlighted in Figs. 1 and 3) using an adjustable-volume single-channel micropipette (Eppendorf Research Plus). LED pulse sequences (Fig. 4) were initiated immediately after sample droplet placement. Data from both oscilloscope channels ($V_{L}$ and $V_{\text{LED}}$) were saved.

### 2.4. Chemical identification via machine learning

#### 2.4.1. Quantity of measurements

Table 1 lists the quantity of sample measurements used for machine learning training and testing. Note that the testing samples are kept independent of training data. Each sample measurement was performed on a new device. This reduces issues with any chemicals remaining on a device surface, and falsely indicating chemical detection on a subsequent measurement. This approach captures device-to-device variability directly within the model. The training/testing sample ratio followed an 80%/20% random selection.

#### 2.4.2. Data pre-processing

All data analysis was performed with a standalone Python script (provided in SI). The script uses pandas, SciPy, and Matplotlib for data handling, numerical fitting, and visualization, respectively (Hunter, 2007; McKinney, 2010; Virtanen et al., 2020). Once all measurements were loaded, any DC offset within each measurement was removed by averaging the first 100 data points, the period before LED illumination, and subtracting that average point by point from the entire pulse set measurement.

**Fig. 5.**
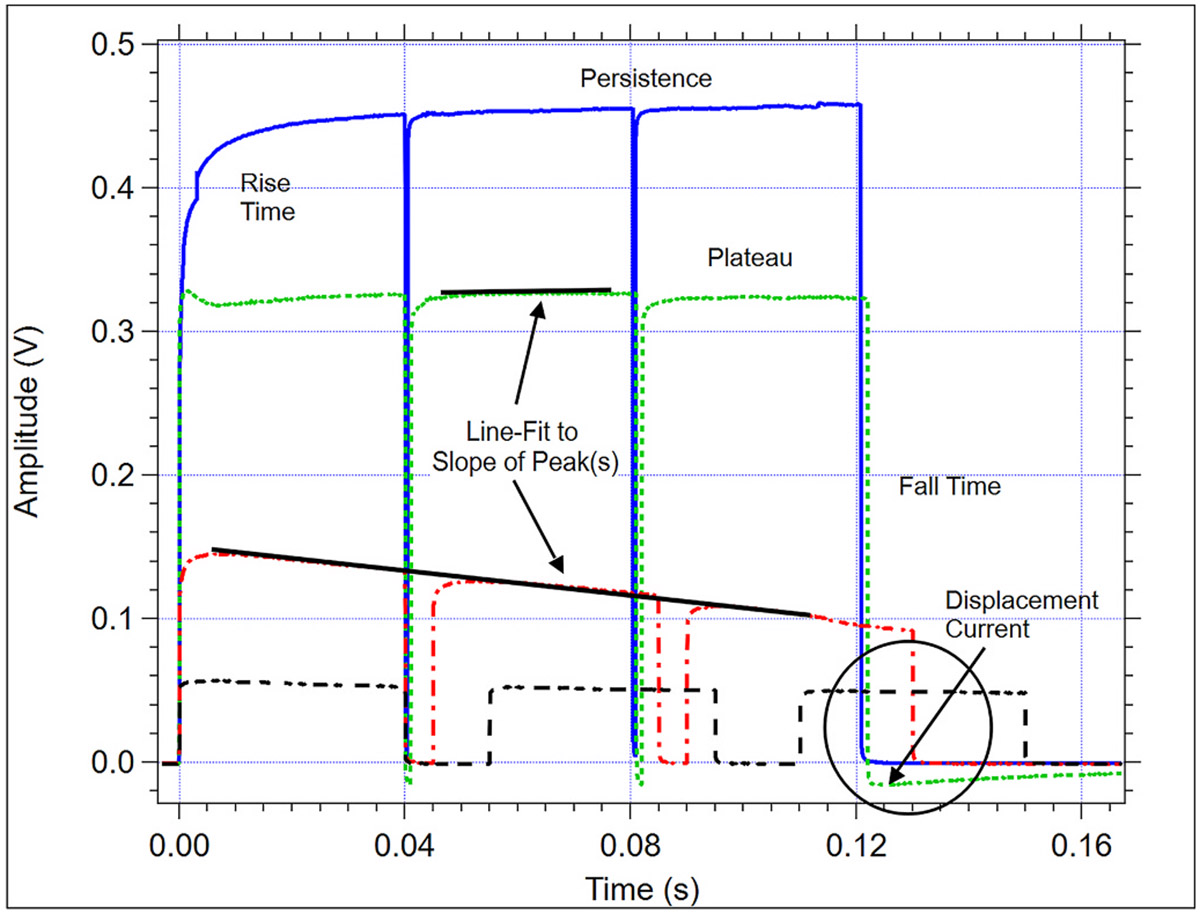
Features of interest to differentiate chemical interactions and kinetics.

Specific regions of interest were identified within each measurement (Fig. 5). These regions were then fit to the equations given in Table 2. These regions are: each pulse rising edge, falling edge, steady state, and peak fit. The steady state fit is a line fit across a single peak, whereas the peak fit is a line fit across the three amplitudes of the steady state regions within a sequence. In order to simplify the fit process, each region received a locally shifted time vector such that the first point of the region was set to time t = 0 through point-by-point subtraction. The mean, standard deviation, minimum, and maximum values of each region were also taken as features.

#### 2.4.3. Model setup and training

The pre-processed dataset was split into training and test groups using an 80 percent stratified shuffle split to preserve class proportions. Chemical identification was performed using a one versus rest (OvR) strategy in which an independent Random Forest (RF) classifier was trained for each chemical. Each classifier evaluates whether its target chemical is present, absent, or inconclusive in a sample, allowing identification in mixed or unknown compositions without forcing a label when none of the trained chemicals are present. Feature selection, threshold determination, and model tuning were carried out using cross-validation within the training set. Detection thresholds were optimized using precision-recall analysis to balance false positives and false negatives, and an inconclusive region was defined to avoid uncertain assignments. Model performance was evaluated using a separate test set that was not used for training. The entire training and testing process was repeated with multiple randomized train–test splits to assess model stability and generalizability. For each trial, a confusion matrix visualizing the model predictions was generated. Aggregated confusion matrices were created by row-normalizing each trial confusion matrix and calculating the mean and standard deviation for each cell. The accuracy of the model predictions is determined using the true positive rates (TPR), true negative rates (TNR), false positive rates (FPR), and false negative rates (FNR) along with the F1 score (F1 = 2 × (precision × recall)/(precision + recall)). Mean, standard deviation, and 95% t-distribution confidence intervals were calculated for model accuracy and F1 scores. Additional details are provided in the SM. Precision-Recall (PR) curves were also generated for each trial. PR curves visualize the model's tradeoff between precision, the measure of how accurate predictions of ‘present’ or ‘true’ are correct, and recall, which is equivalent to the true positive rate. PR curves were selected over other visualizations such as Receiver Operating Characteristic (ROC) curves, which plot the model's true positive rate vs the false positive rate, due to the imbalance between positive and negative cases for each class since the set of negative cases contains all other classes. ROC curves are based on the FPR, which is skewed by the inclusion of true negatives that may inflate the model performance metric, which PR curves avoid. For each PR curve the average precision (AP), or area under curve, is calculated to represent class performance. The more area under the PR curve, the more accurate the model predictions are.

#### 2.4.4. Multi-class RF classifier

For comparison with the OvR framework, an additional multi-class RF classifier was trained using the “argmax” selection rule, which assigns each sample to the class with the highest predicted probability. Although this method is not suited for mixtures or samples that may not contain any trained analytes, it provides insight into feature separability among the classes. The model was trained using the same preprocessing and feature extraction steps as the OvR approach, and its performance was evaluated across multiple randomized splits to assess sensitivity to training data composition.

#### 2.4.5. Total organic fluorine model

In addition to the OvR and simplified classifier described above, a TOF model was developed to evaluate performance for detection of combined TOF, rather than individual chemicals. Through this, the model is trained to learn the features that fluorinated samples share in common instead of focusing on differentiation of structurally similar PFAS. A TOF OvR model was trained in addition to a TOF classifier using the single random forest classifier setup using the same preprocessing, feature selection, and validation procedures described in the preceding sections. The only modification was that during data labeling all PFAS samples were merged into a single group rather than being labeled individually with the other three alcohol classes remaining unchanged.

## 3. Results

### 3.1. ENVIR-OGT structure and operation overview

**Fig. 6.**
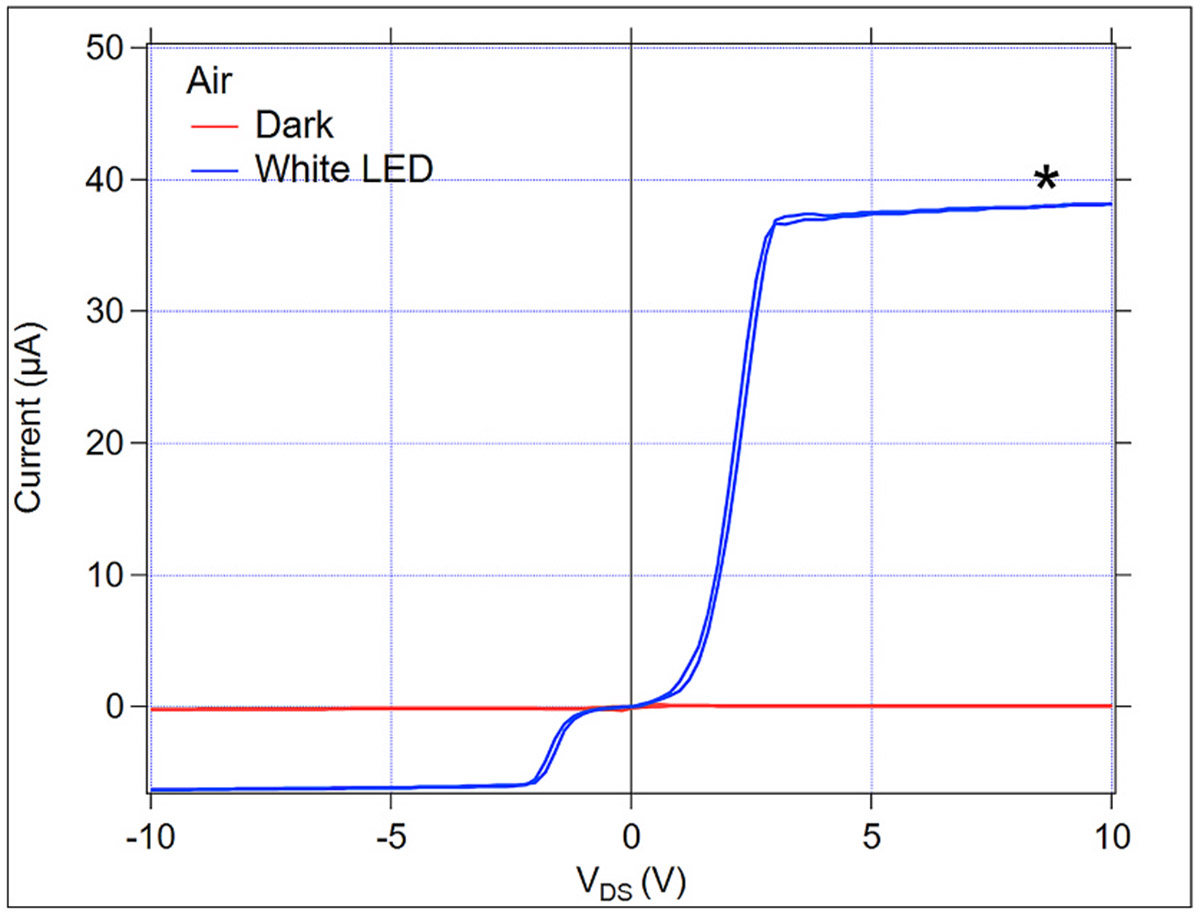
Light and dark I-V curves of a typical ENVIR-OGT measured in air. *Denotes the drain to source bias voltage, 8.2 V.

The ENVIR-OGT is a photogated field-effect transistor fabricated from standard thin films: a floating p-type Si substrate, native SiO_2_ layer, a-Ge_2_Se_3_, and W source–drain electrodes (Fig. 1). Between the electrodes, an open region of a-Ge_2_Se_3_ is left exposed for sample placement and illumination. Illumination during chemical detection occurs close to the drain electrode (Fig. 1(b), circled region). When the device is illuminated with visible light, electron–hole pairs are generated both in the p-Si substrate and within the a-Ge_2_Se_3_ layer. Under these conditions, current flow could have multiple pathways between the source and drain, including: (1) the p-Si/SiO_2_ interface, (2) the a-Ge_2_Se_3_/SiO_2_ interface, and (3) the p-Si bulk (Fig. S3). The resulting *I*–*V* characteristics resemble those of a field-effect device (Fig. 6), and the hysteresis observed in the illuminated *I*–*V* trace is consistent with charge trapping in the a-Ge_2_Se_3_ layer and at the a-Ge_2_Se_3_/SiO_2_ interface. Note in Fig. 6, there is current in the -V direction during illumination as happens when the light is not completely over the drain electrode area; as light is directed closer to the source, carriers are generated near the source region producing an apparent negative current in the negative voltage region. In a-Ge_2_Se_3_, photogenerated carriers likely localize in tail and mobility edge states and as a result, their direct contribution to the steady-state source–drain current is small. However, these trapped charges can influence the transient response, producing the distinct pulse-response features that differentiate chemical analytes.

Application of visible-light pulses likely produces a coupled response in the two photoactive layers. The floating p-Si develops a surface photovoltage (SPV) at the p-Si/oxide interface, while the a-Ge_2_Se_3_ layer undergoes photogating. These processes likely act on different timescales, the rapid SPV rise, and the slower trap-filling dynamics, so the resulting source-drain current carries a rich, analyte-dependent kinetic structure.

To verify that the device response depends on the p-Si and the a-Ge_2_Se_3_ layer, two types of control structures were fabricated: 1) a thermal SiO_2_ layer, instead of a-Ge_2_Se_3_, using Cr electrodes; and 2) a p-Si substrate that replaced the native oxide with Si_3_N_4_, but retained the a-Ge_2_Se_3_ and W electrodes. Neither of these device types showed a measurable response under DC or pulsed illumination conditions, and exhibited no detectable interaction with surface-applied chemicals.

#### 3.1.1. Material selection

The a-Ge_2_Se_3_ layer was selected for its combination of electronic stability, high resistivity in the dark, and compatibility with thin-film processing. Amorphous Ge_2_Se_3_ is photoresponsive, yet maintains extremely low dark current, enabling a large dynamic range under optical excitation. The a-Ge_2_Se_3_ layer provides strong capacitive coupling to the underlying substrate while preserving ultra-low leakage current.

From a practical perspective, the absence of long-range order in the amorphous film contributes to excellent device-to-device reproducibility. This Ge-rich composition has a high dielectric constant of approximately 11.8 (Feltz, 1993) and a high glass-transition temperature of approximately 350 °C which ensures high resistivity and thermal stability during fabrication and in operation. Importantly, the a-Ge_2_Se_3_ layer can be exposed directly to liquid samples and illumination without the need for analyte specific functionalization, allowing the device response to be probed under controlled optical stimulation rather than using equilibrium surface chemistry.

#### 3.1.2. Operation in the dark

With the device biased at $V_{DS}=8.2$ V between the drain and source electrodes, the channel exhibits a dark resistance in the GΩ range. The floating p-Si layer provides no direct current path, and charge transport in the a-Ge_2_Se_3_ channel is limited to thermally activated hopping between localized states. As a result, the film behaves as a glassy insulator. Even with several volts applied across the millimeter-scale gap, the lateral dark current remains in the fA–pA range. This highly resistive baseline state provides a stable reference against which illumination-induced changes can be measured. The native SiO_2_ on p-Si places the silicon surface in depletion, producing upward band bending and a positive surface potential. Because the p-Si is electrically floating and separated from the metal contacts by the oxide, no DC conduction occurs through this layer.

#### 3.1.3. Operation under illumination

Under visible illumination, two coupled photoelectronic processes act together to modulate the effective channel conductance. Light absorbed within the a-Ge_2_Se_3_ layer generates electron–hole pairs, leading to partial filling of shallow traps and increased carrier population in band-tail and near-mobility-edge states, thereby enhancing the film's photoconductivity. At the same time, a portion of the incident light penetrates the thin chalcogenide film and reaches the p-Si substrate, where it generates carriers that modify the space-charge field at the Si/SiO_2_ interface. The resulting SPV reduces the upward band bending of the p-Si surface and capacitively gates the a-Ge_2_Se_3_ channel through the series combination of the silicon space-charge capacitance, the native oxide, and the chalcogenide film. These two effects, the increased photoconductivity of a-Ge_2_Se_3_ and the SPV-induced gating of the underlying p-Si, combine to produce the pronounced increase in drain current observed under illumination. When the light is removed, recombination in the silicon and the release of carriers from traps in a-Ge_2_Se_3_ return the device to its dark state, with the persistence time governed by the energy distribution and occupancy of localized trap states.

Optical excitation produces transient changes in the measured source–drain current during illumination, followed by a relaxation after light removal. The resulting current waveform contains features spanning multiple timescales, including changes at illumination onset, evolution during each light pulse, and relaxation following termination of optical excitation. When a liquid sample is present, these features differ reproducibly from the air baseline under otherwise identical measurement conditions.

Distinct regions of the transient waveform were analyzed following each light-pulse sequence, as illustrated in Fig. 5 and quantitatively defined by the fitting functions in Table 2. These waveform-derived features provide a compact and reproducible description of the device response across multiple timescales and form the basis for the comparative and machine-learning analyses presented in the following sections.

### 3.2. Pulse sequence test results

#### 3.2.1. Neat alcohols

**Fig. 7.**
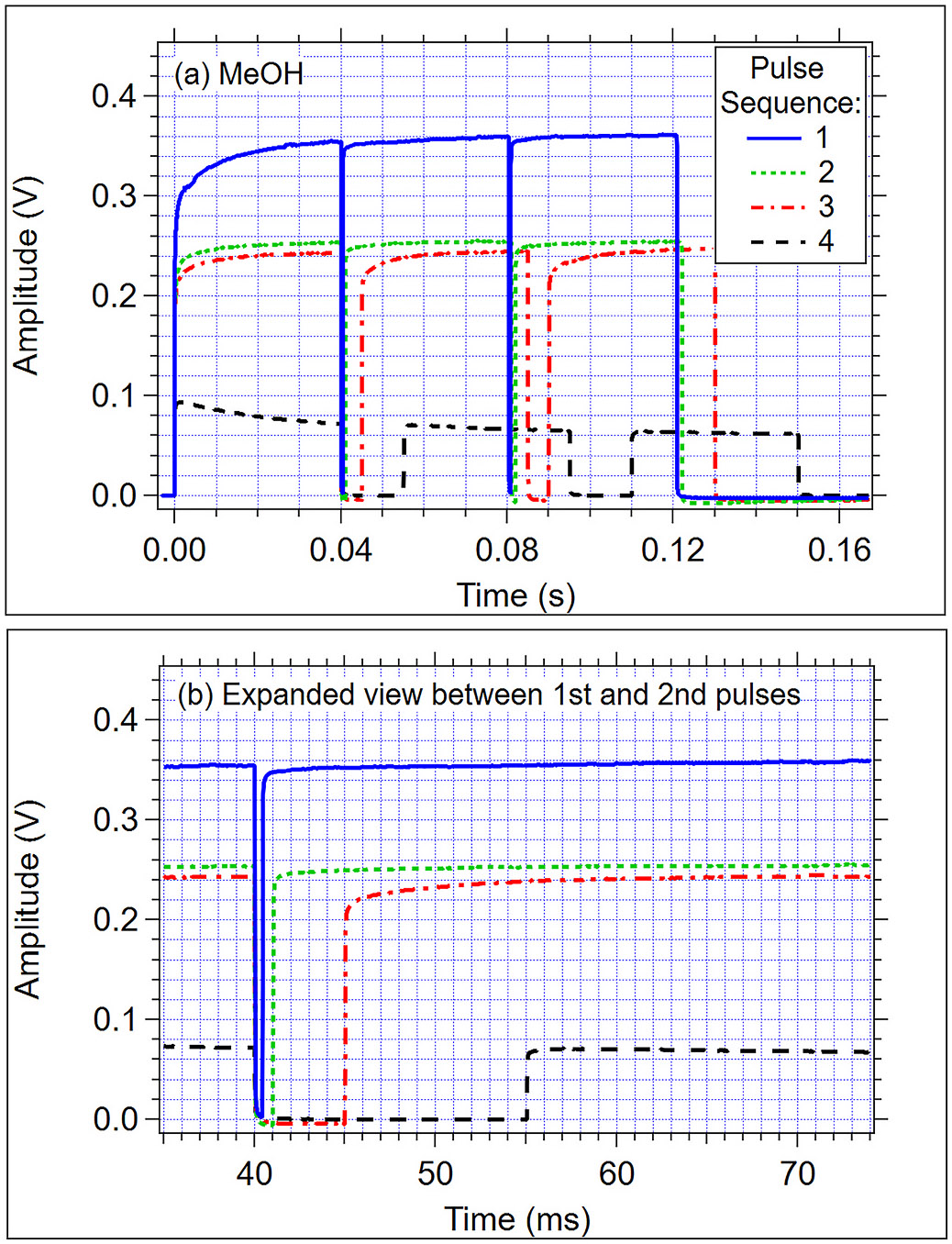
Response to light pulse sequences in presence of MeOH. (a) full pulse set for each sequence. (b) expanded view of the first pulse falling edges for each sequence.

**Fig. 8.**
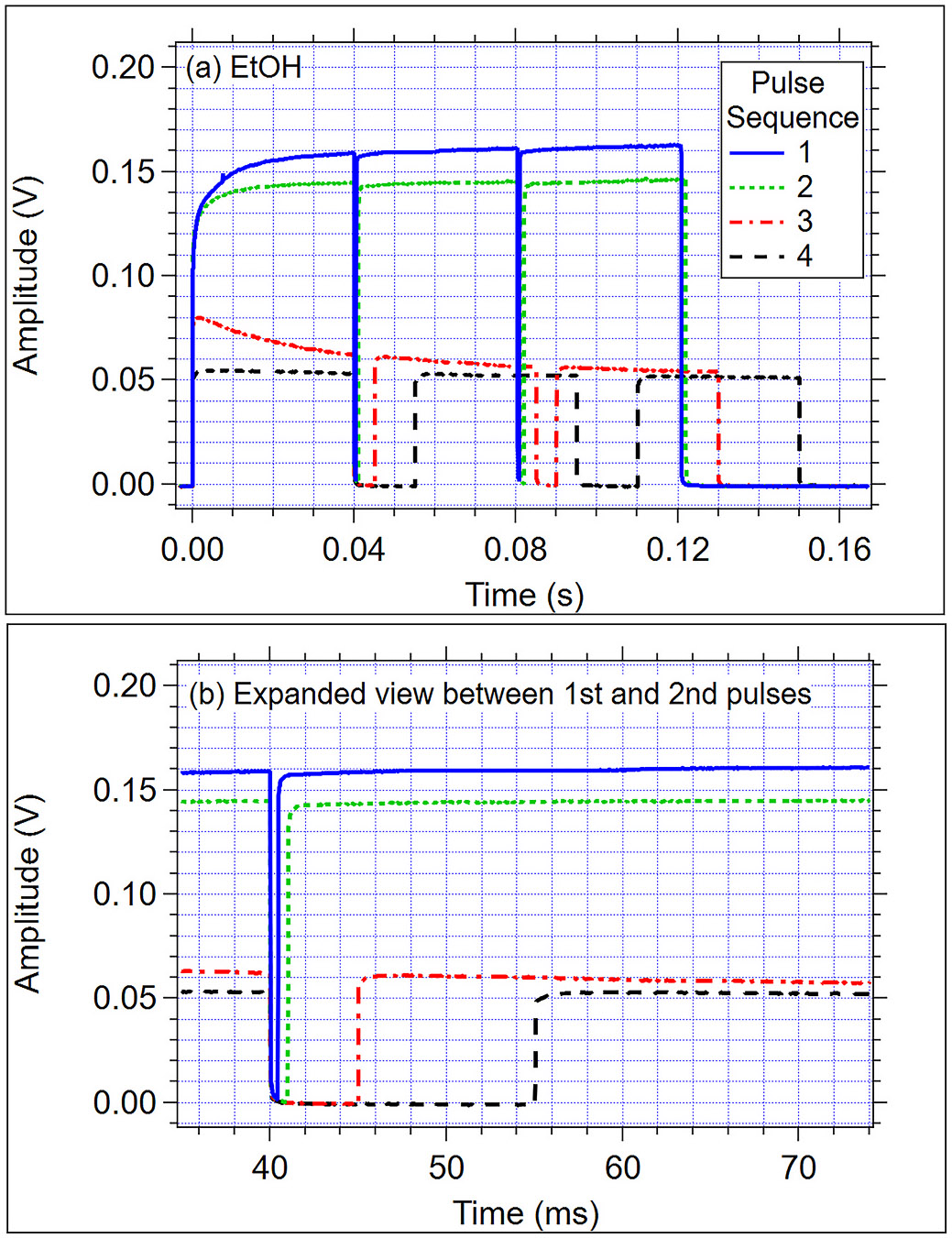
Response to light pulse sequences in presence of EtOH. (a) full pulse set for each sequence. (b) expanded view of the first pulse falling edges for each sequence.

**Fig. 9.**
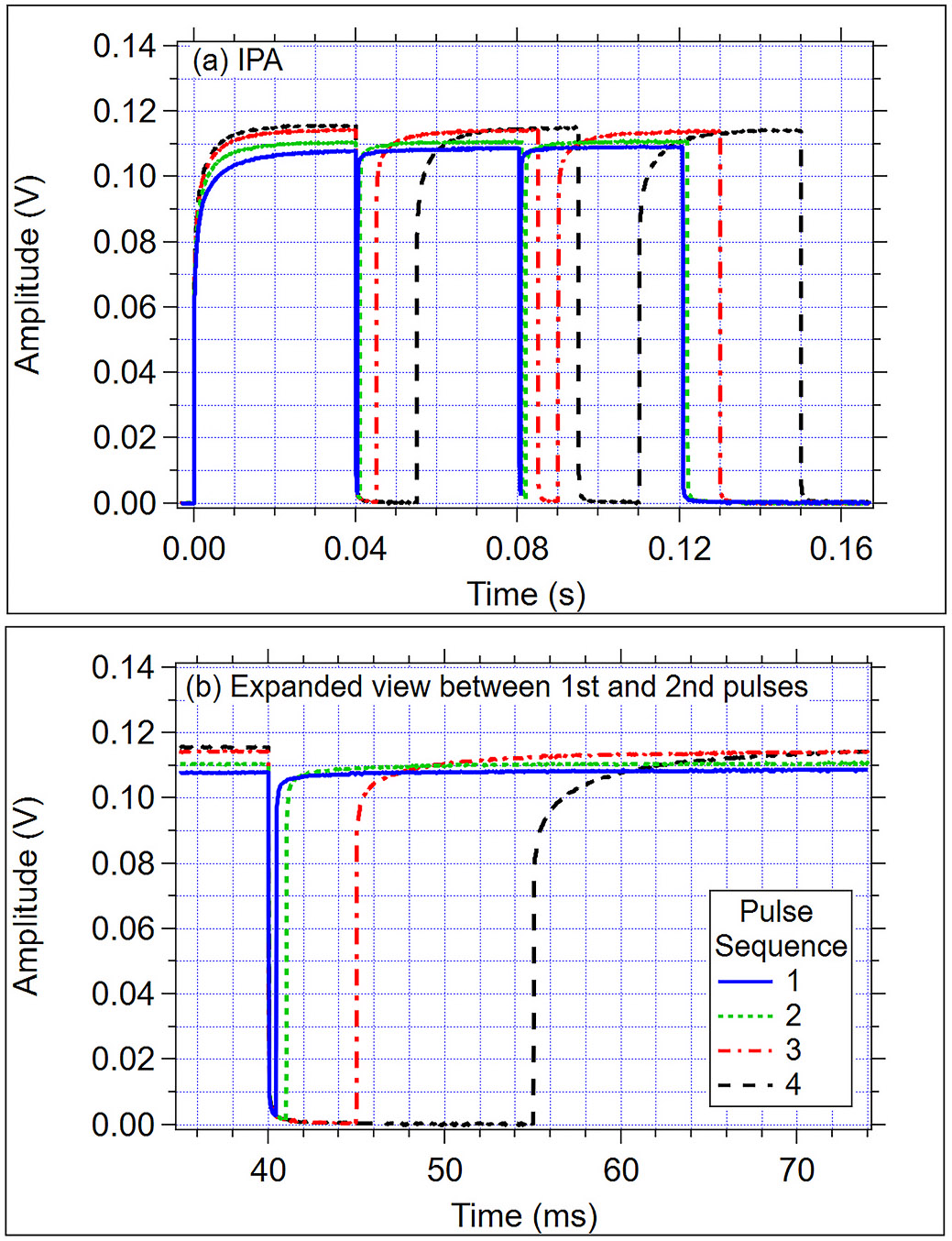
Response to light pulse sequences in presence of IPA. (a) full pulse set for each sequence. (b) expanded view of the first pulse falling edges for each sequence.

The neat alcohol measurement classes each showed a clear distinction between the features of interest (Figs. 7-9) and across the four pulse sequences. The differences manifest in the rise and fall times, pulse amplitudes, decay as a function of pulse number, and in the comparison of these features between different pulse sequences.

For example, a typical MeOH measurement includes a first pulse sequence that exhibits a substantially larger amplitude than the second pulse sequence (Fig. 7). In contrast, in a typical EtOH sample the second sequence amplitude remains only slightly reduced from the first pulse sequence amplitude (Fig. 8). The typical IPA response (Fig. 9) differs markedly from those of MeOH and EtOH. Across all four pulse train sequences, the amplitude remains nearly constant, with a notable exception of a slight increase in peak height as the sequence progresses.

Another distinguishing feature among the tested alcohols is the presence or absence of a displacement current following the last pulse, most easily seen (when present) after the final pulse of a sequence (see Fig. 5). This feature is slightly visible after the last pulse of sequence 2 for the neat MeOH sample (Fig. 7).

These sequence-dependent and time-domain differences were reproducible across multiple devices, indicating that the observed features reflect the sensing approach rather than device-specific variability. The resulting transient signatures provide sufficient contrast for reliable differentiation among methanol, ethanol, and isopropanol using the waveform-derived features described in Section 2.4.

#### 3.2.2. PFAS

Each PFAS sample was independently measured using the same pulse techniques as the alcohol measurements.

**Fig. 10.**
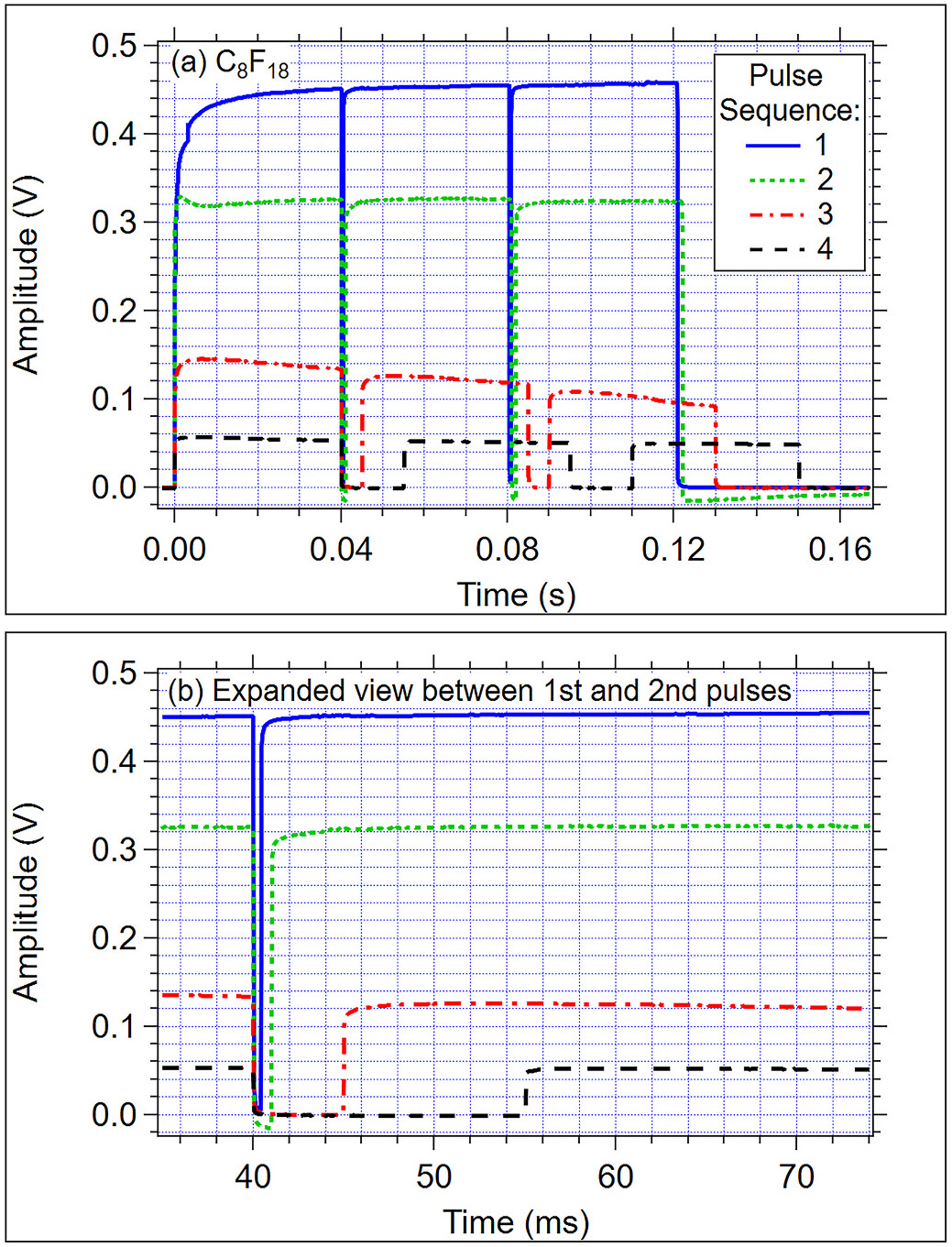
(a) Typical pulse response of C_8_F_18_ at 1 ppm in MeOH. (b) Expanded view of first falling edge.

C_8_F_18_ is nonpolar, lacking a polar headgroup. In the ENVIR-OGT measurements, it produces a transient response that differs from the other analytes tested. In many samples, a displacement-current transient appears during the second pulse sequence (Fig. 10), and the subsequent sequences often show a rapid reduction in signal amplitude and a characteristic within-pulse decay by sequence 3. By sequence 4, the response approaches the baseline (no-analyte) behavior. The origin of these features is not yet established, but they provide a practical fingerprint for identifying C_8_F_18_ in this measurement framework. Representative data for PFPeA and PFPrA are provided in SM, Fig. S3.

### 3.3. Machine learning chemical identification

**Fig. 11.**
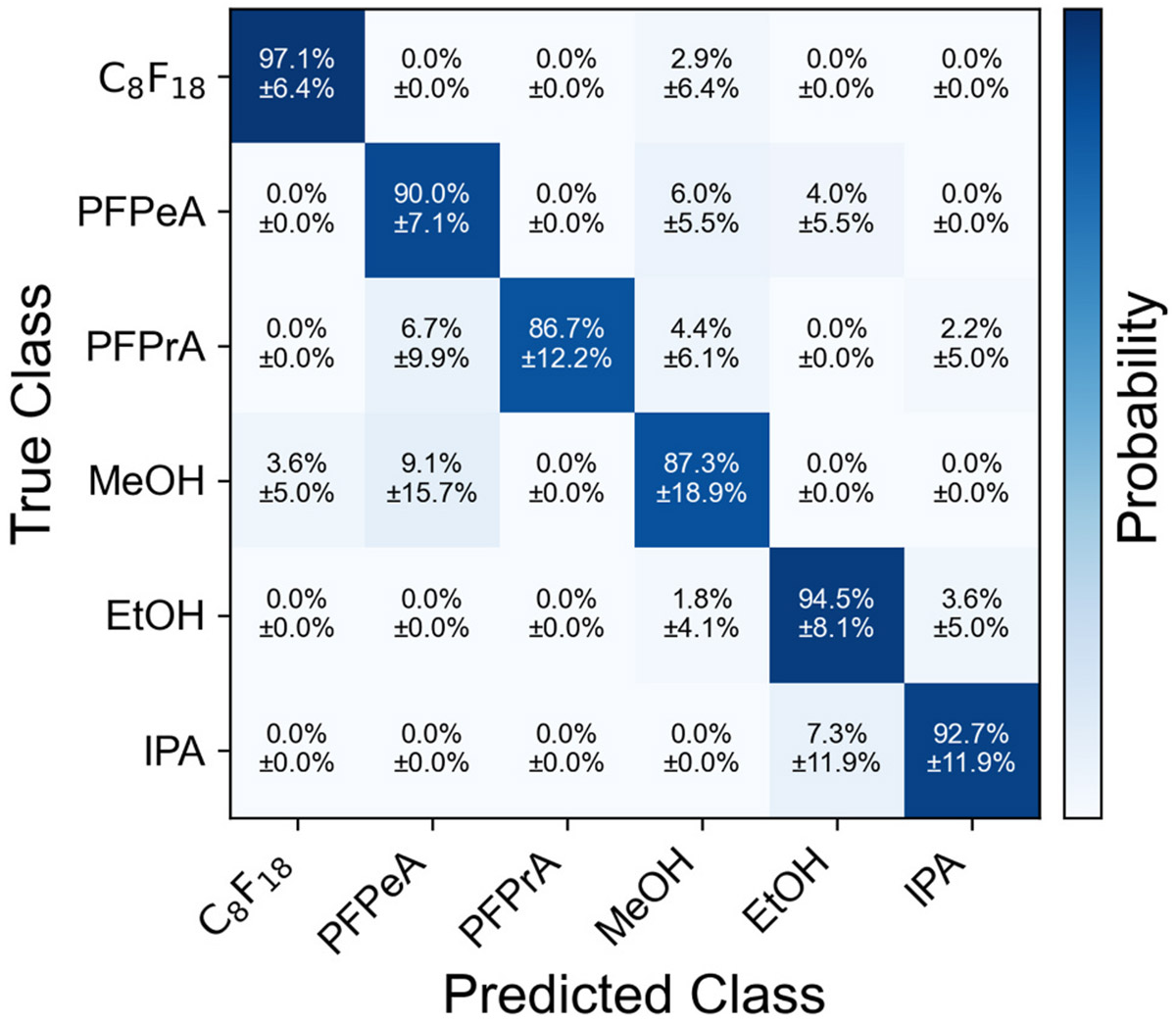
Confusion matrix for the multi-class Random Forest classifier applied to the six chemical classes aggregated across all trials. Each cell reports probability ± standard deviation. See Table 1 for the number of training and test samples.

**Fig. 12.**
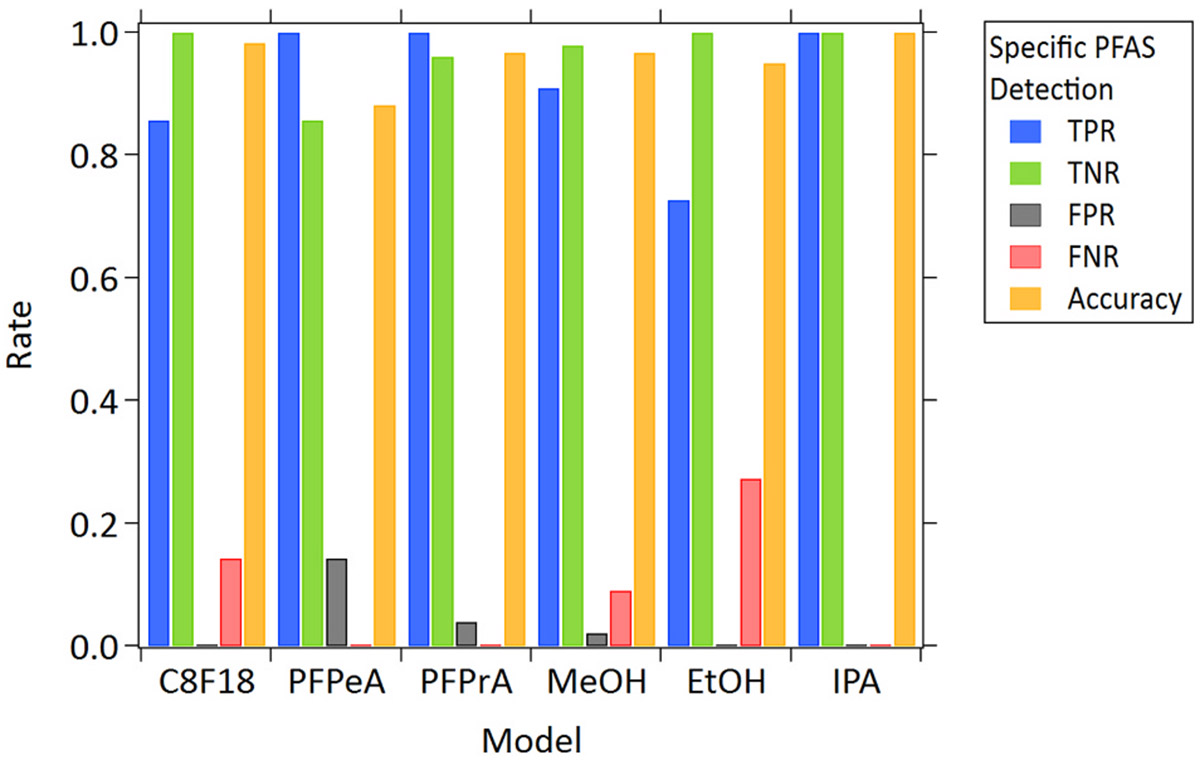
Detection-rate metrics (TPR, TNR, FPR, FNR, and accuracy) for the one-versus-rest Random Forest classifiers applied to the six chemical classes. See Table 1 for the number of training and test samples.

**Fig. 13.**
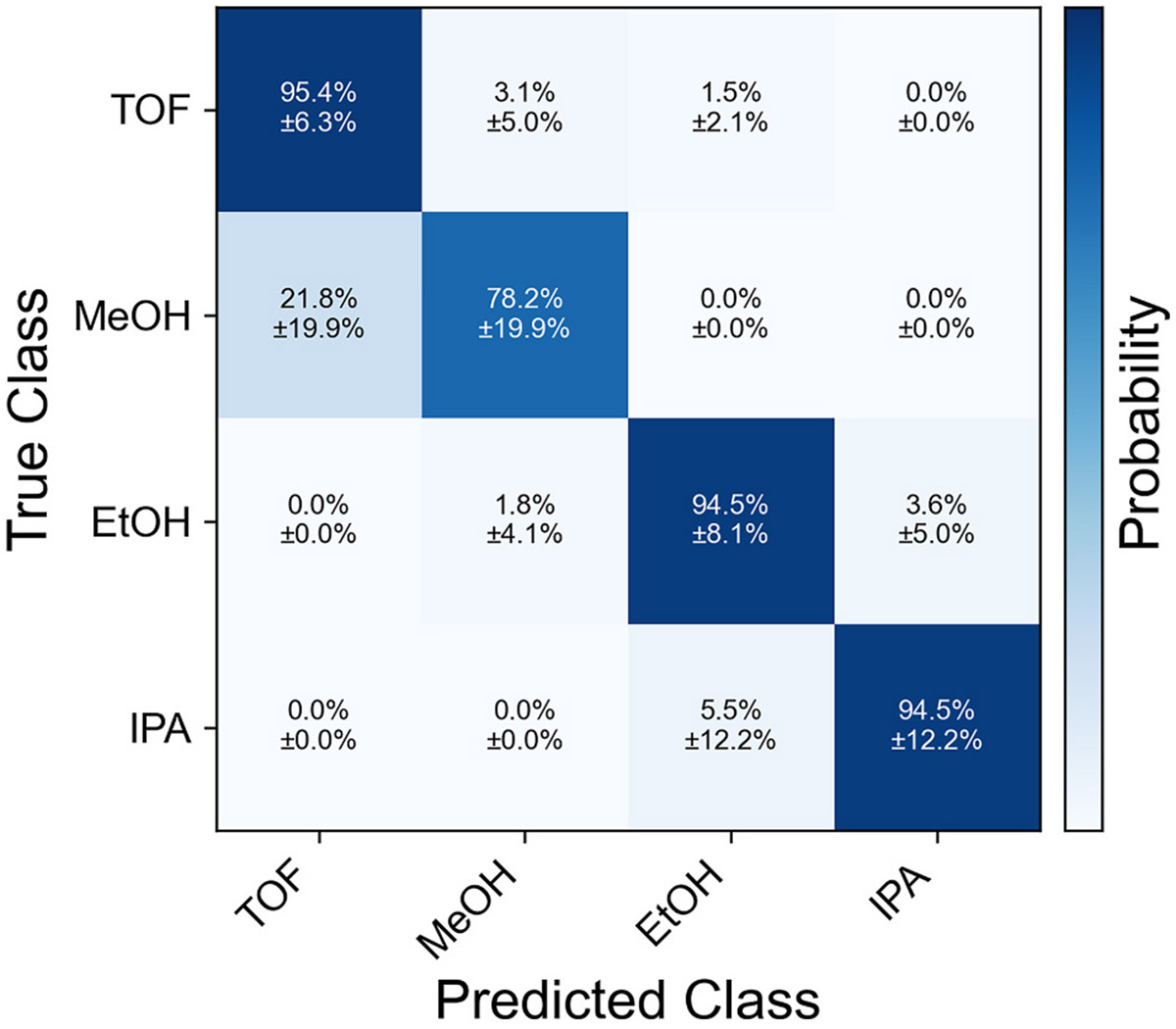
Confusion matrix for the multi-class Random Forest classifier distinguishing TOF (all PFAS merged) from the three alcohol classes. Results shown are aggregated across all trials. Each cell reports probability ±standard deviation. Per trial data are provided in SM, Fig. S8. See Table 1 for the number of training and test samples.

**Fig. 14.**
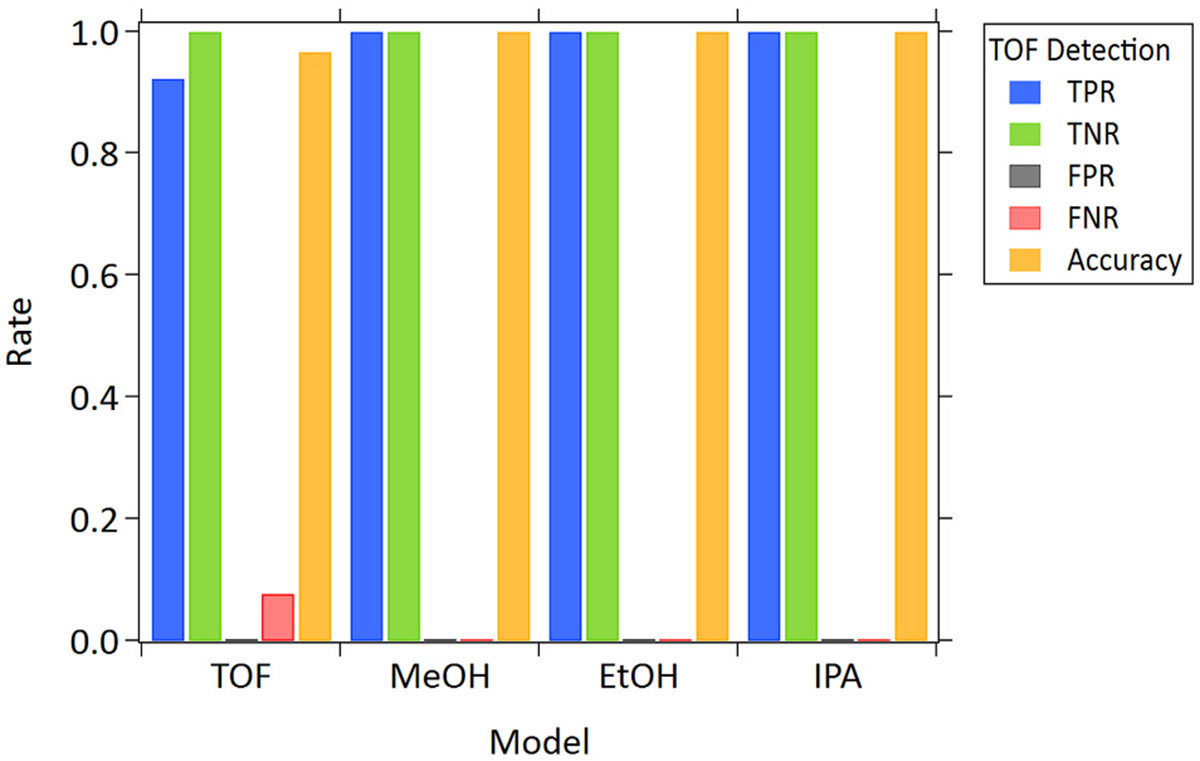
Detection-rate metrics for TOF and alcohol detection using the OvR classifier. Results correspond to the representative case shown in Fig. 12. See Table 1 for the number of training and test samples.

Machine learning was applied to classify the six chemical classes measured in this study: the three neat alcohols (MeOH, EtOH, IPA) and the three PFAS analytes (C_8_F_18_, PFPeA, PFPrA). Each measurement was performed on a new ENVIR-OGT device, ensuring independence of training and test data and capturing device-to-device variability directly within the model. After preprocessing and feature extraction, the dataset was randomly divided into training and test sets using an 80/20 split, and the process was repeated multiple times to assess model stability. One-versus-rest metric charts for representative train–test splits and aggregated confusion matrices are shown in Figs. 11-14; full statistics for all samples across repeated randomized splits are provided in the SM, Fig. S6–S9.

Fig. 11 presents the confusion matrix aggregated across all five trials, using the multi-class Random Forest classifier. PFAS analytes C_8_F_18_ and PFPrA were correctly classified in all samples in the test set. PFPeA was correctly classified in 9 out of 10 samples. Among the alcohols, MeOH and IPA showed perfect classification, while EtOH was correctly identified in 9 of 11 samples.

Detection-rate performance for each chemical was evaluated using the OvR RF classifiers with threshold-based present/absent decisions. These rates for the representative split are shown in Fig. 12, with all classes exhibiting accuracies above 90%. Comparable performance was obtained across additional randomized splits, as summarized in the SM.

To evaluate fluorine-specific detection, the PFAS analytes were grouped into a single TOF class and classified against the three alcohols using the same feature-extraction and preprocessing pipeline. The multi-class TOF confusion matrix for the representative split is shown in Fig. 13 and demonstrates clean separation of fluorinated from non-fluorinated samples. The corresponding OvR detection-rate metrics (Fig. 14) show accuracies exceeding 95% for TOF and 100% for the alcohol classes.

**Fig. 15.**
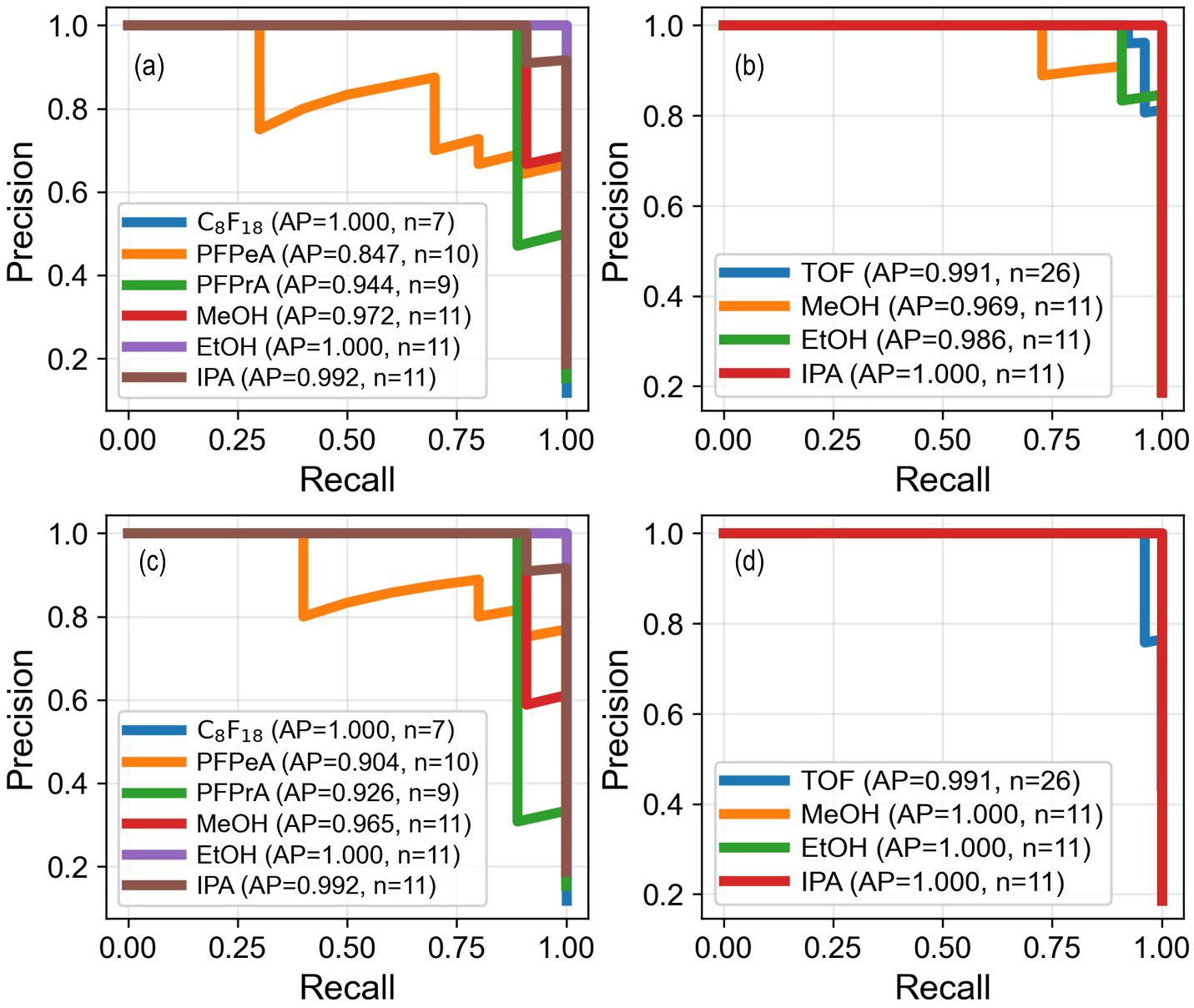
Representative precision-recall curves for classifiers: (a) Separated PFAS OvR, (b) TOF OvR, (c) Multi-class RF, and (d) TOF RF.

The precision-recall curves generated for each model were consistently high across each randomized trial, with average precision values consistently near 1.0 across classes, except for a small number of trials where PFPeA or PFPrA APs were near 0.70. Representative PR curves for each model are shown in Fig. 15.

### 3.4. Summary of classification performance

Two classification strategies were evaluated to assess the discriminative power of the ENVIR-OGT transient features: (1) a multi-class RF model and (2) OvR RF framework.

The multi-class RF classifier assigns each sample directly to one of the trained analyte classes using a highest-probability (argmax) decision rule. In this approach, all classes are considered simultaneously, and each waveform is labeled as the single most likely chemical. This method is useful for evaluating overall separability among the analytes but is not designed for threshold-based present/absent decisions or for mixture detection scenarios.

In contrast, the OvR RF framework trains an independent binary classifier for each analyte against all others. Each classifier outputs a probability that a given sample belongs to its target class. This structure enables threshold-based present/absent decisions and allows calculation of detection-rate metrics such as TPR, TNR, FPR, FNR, and overall accuracy for each analyte individually.

Using the multi-class RF model, classification accuracies exceeding 95% were obtained for PFAS and 100% for the alcohol classes in representative splits with an average accuracy of 91.2% ± 4.0% (standard deviation), 95% t-confidence interval: [86.2%, 96.2%], across all trials. The macro F1 score was calculated at 91.3% ± 4.4% (SD), 95% t-CI: [85.9%, 96.7%], which indicates that the classifier's performance is consistently high across classes with well-balanced precision and recall across trials. The model successfully distinguished individual PFAS species, including the ultra-short-chain PFPrA, despite structural similarity. These results confirm that the extracted transient features provide sufficient separability for direct multi-class identification. Similar performance was observed for the TOF RF model with an average accuracy of 91.9% ± 5.5% (SD), 95% t-CI: [85.0%, 98.8%], and macro F1 score of 91.0% ± 6.3% (SD), 95% t-CI: [83.2%, 98.8%], across trials.

The OvR RF framework further demonstrated strong and consistent performance across analytes. Detection-rate metrics (Figs. 12 and 14) show argmax accuracies above 90% for all individual classes, with the TOF classifier achieving greater than 95% argmax accuracy.

By merging all PFAS samples into a single TOF class, the model was trained to identify shared fluorination-related waveform features rather than differentiating individual PFAS structures. Clean separation between fluorinated and non-fluorinated samples was observed (Fig. 13).

Because OvR does not force a single label for every sample, the model is evaluated using coverage, the fraction of samples for which the model makes a decisive prediction (exactly one class identified as present), and abstention accuracy, the accuracy computed conditional on those decisive predictions. Together, these metrics quantify the tradeoff between how often the OvR model is willing to make a prediction and how accurate those predictions are when it does. The separated PFAS and TOF OvR models achieve abstention accuracies of 95.9% ± 4.2% at 91.5% ± 3.8% coverage and 94.8% ± 4.1% at 83.1% ± 2.7% coverage, respectively. These figures show that the OvR models successfully learn to abstain from ambiguous samples while maintaining high accuracy when they commit to a ‘present’ label.

Feature ranking was also performed for the RF classifier models to quantify what regions and features were most significant in model prediction and how the ranking of these regions differed between chemicals. The top three features per chemical classifier from a representative trial are listed in Table 3. Random Forest impurity-based feature importance was used to rank features in the OvR analysis. Because each chemical is modeled by a separate one-vs-rest Random Forest classifier, feature importance magnitudes are not strictly comparable across chemicals. Therefore, cross-chemical interpretation focuses on the relative ranking and occurrence of the top features. Across both models, the highest-ranked features were dominated by simple summary statistics such as minimum and maximum amplitude rather than fitted model parameters. This indicates that much of the chemical discriminative information in the ENVIR-OGT transient response is captured by low dimensional descriptors of region amplitude and low-resolution temporal shape, suggesting that the transient response is highly compressible while still retaining chemical specific information.

## 4. Discussion

Because the applied optical pulse sequence is identical across measurements, differences in the ENVIR-OGT current response arise from how each analyte solution interacts with the device surface within the illuminated region. While this study does not quantify the physical mechanisms responsible for specific features, the data indicate that the chemical environment modifies the transient response in a systematic and reproducible manner, and the potential physical mechanisms present in this type of device can be qualitatively associated with specific features. For example, a rapid photoinduced charge redistribution may correlate with a fast current rise time response of the ENVIR-OGT. The persistence and fall time responses can be associated with charge trapping and de-trapping, and charge redistribution when light is removed. A displacement current typically indicates an abrupt change in interfacial potential.

A key outcome of this work is that this simple thin-film architecture can distinguish structurally similar PFAS, including short- and ultra-short-chain species at the tested 1 ppm concentration, as well as differentiate between common alcohols. The unique measured features for different analytes are a result of the set of four temporally separated optical pulse sequences. These features allow machine-learning classifiers to separate analytes even when their steady-state electrical responses are similar. For example, the rise time feature most clearly differentiates PFAS from methanol, while peak amplitude and fall time features are ranked highest in the TOF model. These trends suggest that fluorination influences both the initial transient charging and subsequent relaxation dynamics. Broader generalization to other fluorinated compounds will require targeted validation.

The generation of analyte-specific kinetic fingerprints through designed light-pulse sequences was inspired by magnetic resonance spectroscopy, where carefully structured microwave or RF pulse timing selectively probes distinct electron or nuclear interactions within a chemical system. The sensing strategy is conceptually similar to multi-dimensional spectroscopies, where varying the timing between pulses allows different relaxation pathways and interaction mechanisms to be selectively emphasized. In the ENVIR-OGT, the temporal spacing and duration of optical pulses similarly govern which photoinduced and trap-mediated processes dominate the observed response, allowing distinct kinetic regimes to be emphasized. More broadly, the idea that temporal pacing acts as a consequential design variable influencing the information retrieved has been recognized across multiple disciplines. For example, studies of time-compressed media consumption have shown that playback speed modulates which cognitive and affective processes shape measurable outcomes (Yueh et al., 2025). In this context, pulse timing must be selected to balance measurement throughput with information content. More broadly, these results show that optically gated transients can retrieve chemically specific information using a simple thin-film device architecture. The ENVIR-OGT provides a compact, low-power platform in which chemical environments manifest as predictable variations in transient electrical behavior, supporting data-driven identification. The demonstrated classification of individual PFAS and TOF highlights the potential of this approach for extending transistor-based sensing to a wider range of analytes and motivates further exploration of pulse schemes, illumination conditions, and material systems to enhance selectivity and robustness.

## 5. Conclusion

This work demonstrates that transient optical gating in a thin-film transistor can extract chemically specific information without relying on chemical surface modifications or reference electrodes. By applying designed light-pulse sequences, the ENVIR-OGT produces reproducible kinetic signatures that vary systematically with the chemical environment. These signatures support machine-learning classification of common alcohols as well as individual PFAS, including short- and ultra-short-chain species, and TOF in simple solvent-prepared samples. The results show that chemical identification can emerge from illumination-driven transient responses in a minimal device architecture, providing a compact and generalizable sensing strategy that broadens the functional scope of thin-film-transistor sensors.

Although the present study focused on solvent-prepared samples in methanol to establish controlled chemical differentiation, preliminary measurements in aqueous solutions indicate that the ENVIR-OGT maintains strong differentiation performance across solvent environments. The primary observed difference is a modest increase of rise and fall time constants in water relative to methanol, consistent with increased dielectric screening and more rapid interfacial charge redistribution in higher-polarity media. Solvent polarity and adsorbed interfacial water are expected to influence the balance between rapid photoinduced processes and slower trap-mediated relaxation components by modifying interfacial electrostatics and surface state occupancy. However, the pulse-sequence framework enables dynamic adjustment of temporal probing windows, allowing the measurement protocol to be tuned as needed to emphasize distinct kinetic regimes under varying solvent conditions.

Environmental water matrices introduce additional variables, including dissolved ions, variable pH, and organic constituents, which may alter surface electrostatics and transient behavior. Systematic evaluation of these matrix effects and direct testing with field-collected environmental samples represent important next steps toward real-world deployment. Future studies will explore detection limits, chemical mixtures, aqueous and complex solvents, wavelength-dependent excitation, adaptive pulse-sequence designs, and expanded environmental testing to further enhance selectivity, robustness, and applicability across diverse sensing scenarios. Additionally, future studies will investigate the feasibility of device re-use.

## Supplementary Material

1

## Figures and Tables

**Table 1:** Train and test sample quantity.

Chemical	Total Number of Devices/ Samples	Number of Train	Number of Test
IPA	54	43	11
Ethanol	54	43	11
Methanol	54	43	11
C_8_F_18_	33	26	7
PFPeA	52	42	10
PFPrA	45	36	9

**Table 2:** Fit equations for features of interest.

Region	Equation
Rising Edge	$y(t)=c+a_{1}\left(1-e^{-\frac{t}{\tau_{1}}}\right)+a_{2}\left(1-e^{-\frac{t}{\tau_{2}}}\right)+\;a_{3}\left(1-e^{-\frac{t}{\tau_{3}}}\right)$
Falling Edge	$y(t)=c+a_{1}e^{-t∕\tau_{1}}+a_{2}e^{-{\left(\frac{t}{\tau_{1}}\right)}^{\beta }}$
Steady State and Peak Fit	y(t)=at+b

Note: all variables except for time, t, are resultant fit parameters.

**Table 3:** Representative model top three feature importances.

Rank	1		2		3	
Chemical	Feature	Importance	Feature	Importance	Feature	Importance
TOF	Pulse set 4 peaks region minimum	0.026311	Pulse set 4 fall region 1 minimum	0.025604	Pulse set 2 rise region 1 mean	0.024878
C_8_F_18_	Pulse set 1 rise 1 region minimum	0.043811	Pulse set 1 rise region 3 mean	0.042521	Pulse set 1 steady region 2 mean	0.033985
PFPeA	Pulse set 1 fall region 3 maximum	0.034008	Pulse set 2 steady region 3 maximum	0.032479	Pulse set 1 rise region 3 maximum	0.029892
PFPrA	Pulse set 3 fall region 3 minimum	0.054952	Pulse set 1 fall region 2 minimum	0.051632	Pulse set 1 fall region 1 minimum	0.045661
MeOH	Pulse set 1 fall region 1 minimum	0.062838	Pulse set 1 rise region 3 minimum	0.049535	Pulse set 1 fall region 2 minimum	0.044158
EtOH	Pulse set 4 steady region 1 minimum	0.057111	Pulse set 4 steady region 3 minimum	0.055003	Pulse set 4 peaks region minimum	0.053768
IPA	Pulse set 1 peaks region minimum	0.089004	Pulse set 1 steady region 2 minimum	0.085599	Pulse set 1 steady region 3 minimum	0.083125

## Data Availability

Data will be made available on request.

## Appendix A. Supplementary data

Supplementary data to this article can be found online at https://doi.org/10.1016/j.biosx.2026.100784.

## Abbreviations

ENVIR-OGT: Environmental Optically Gated Transistor
EtOH: ethanol
F1: harmonic mean of precision and recall
FET: field-effect transistor
FNR: false negative rate
FPR: false positive rate
IPA: isopropyl alcohol
LED: light-emitting diode
MeOH: methanol
OvR: one-versus-rest
PFAS: per- and polyfluoroalkyl substances
PFPeA: perfluoropentanoic acid
PFPrA: perfluoropropionic acid (also referred to as pentafluoropropionic acid)
RF: random forest
ROC: Receiver Operating Characteristic
SM: supplementary material
SPV: surface photovoltage
TOF: total organic fluorine
TPR: true positive rate
TNR: true negative rate
