# Supplementary material for: A novel optically gated thin-film transistor sensor for real-time chemical differentiation using machine-learning analysis
Source: Biosens Bioelectron X. Author manuscript; Available in PMC 2026 Jul 9. (PMC13340560; doi:10.1016/j.biosx.2026.100784)
Supplement: 1 [file NIHMS2182285-supplement-1.pdf]

Supporting Information

A Novel Optically Gated Thin-Film Transistor Sensor for Real-Time Chemical Differentiation Using Machine-Learning Analysis

Authors: Lukas M. Crockett, Jacob Jackson, Tucker P. Gratton, Jenée D. Cyran, Bamidele Omotowa, Kristy A. Campbell

S1. Device Fabrication

To fabricate the OGTs a p-Si 8” wafer (1-100 Ω-cm, Microsil, LLC Silicon Services) was cleaved into ~ 3.5” x 3.5” pieces. The pieces were cleaned with an IPA rinse and allowed to air dry before deposition of the OGT gate layer. A layer of native SiO_2 layer was present on the Si surface.

An AJA International ATC Orion 5 UHV Magnetron sputtering system and a Ge_2Se_3 target (from Process Materials, Inc., 2” dia. x 0.125” thick, RF sputtered) were used to sputter deposit the top channel layer to a thickness of approximately 200 Å. W electrodes (350 Å) (or Cr electrodes) were sputter deposited on the surface of the gate layer using a shadow mask (OSH Stencils) to define the source and drain electrodes (Figure S1).

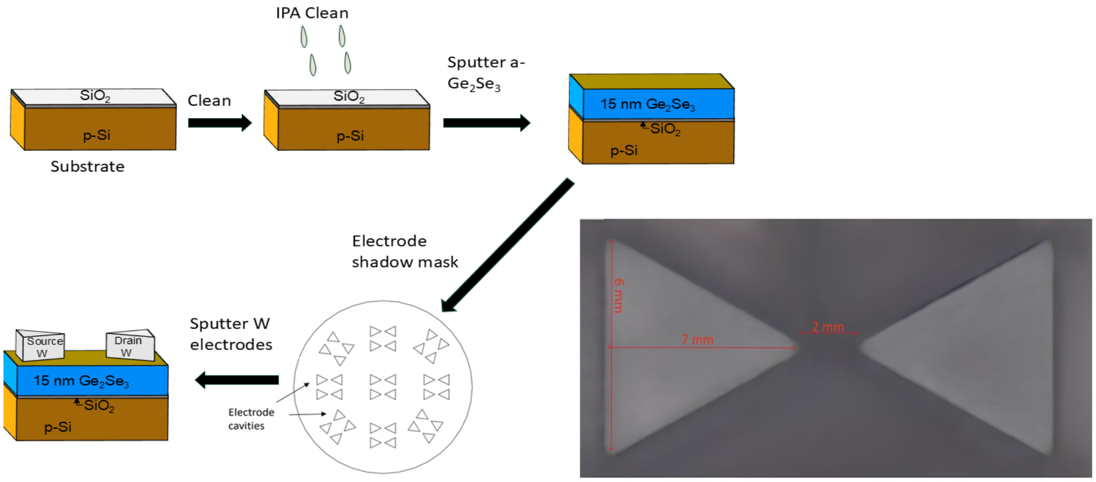

Figure S1. (left) ENVIR-OGT fabrication steps. (right) photograph of one ENVIR-OGT device

S2. Electrical characterization

ENVIR-OGT devices were electrically tested within a fume hood (Fig. S2) for safety, in the event the measurement process produced volatile by products.

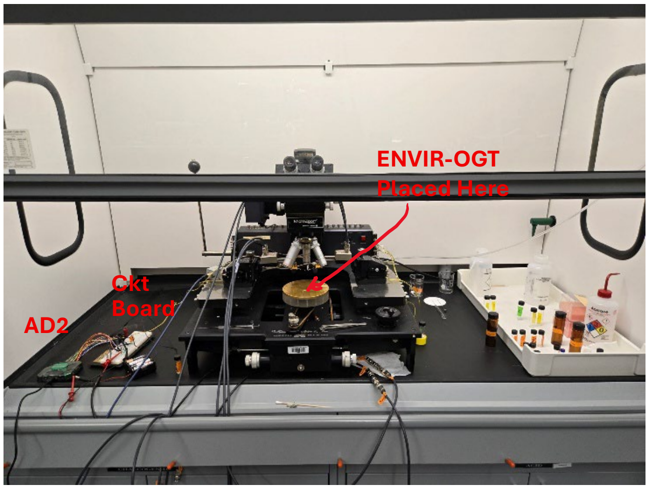

Figure S2. The probe station set up for testing in the fume hood. The battery and pulse driving/data collecting unit (AD2) is on the left side along with a circuit board containing the R_L and $R_{Limiter}$ resistors next to it. The ENVIR-OGT device is placed on the Micromanipulator chuck for testing. Note that the Micromanipulator is not necessary, but is used to simplify the experimental data collection.

The electrical response for the C_8F_{18} sample is shown in the manuscript. The pulse response for PFPeA and PFPrA are shown in Fig. 3a and 3b.

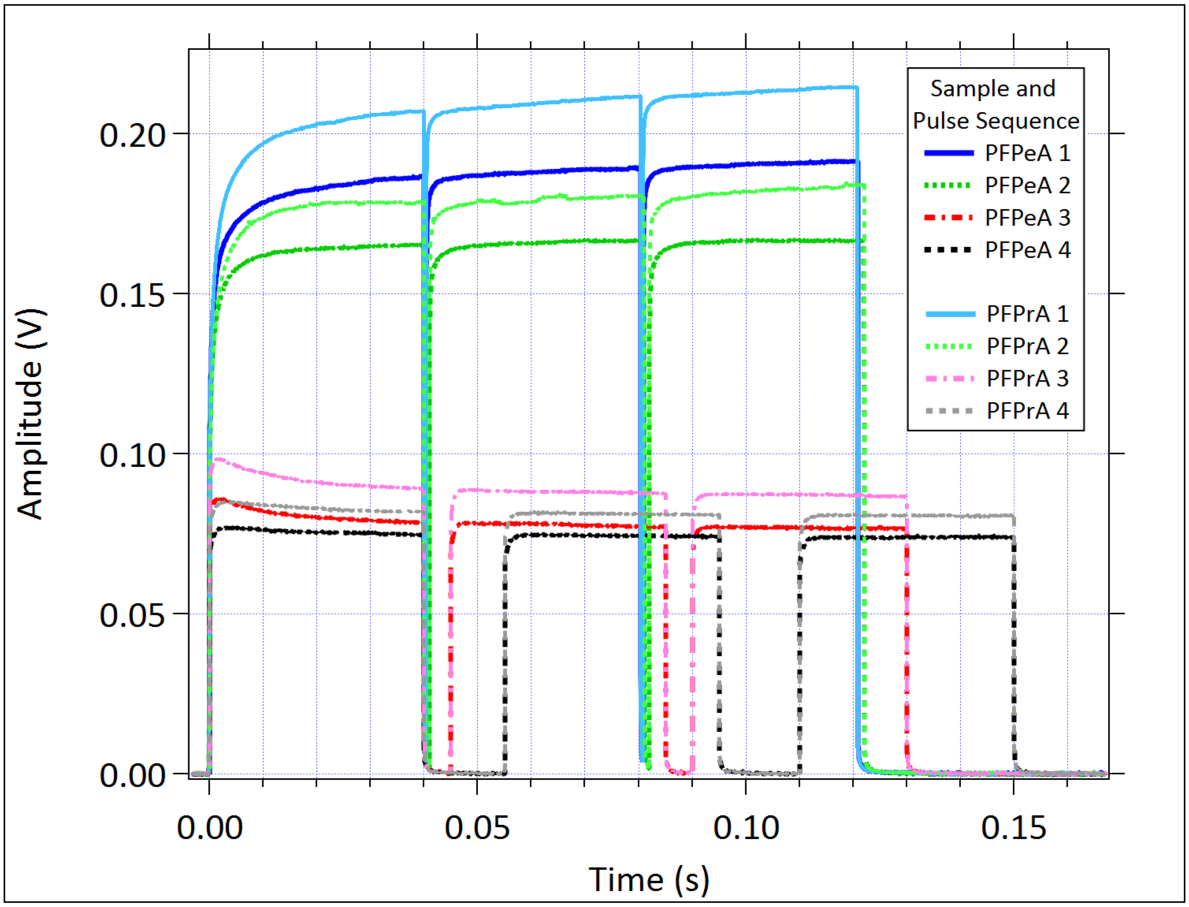

Figure S3a. Pulse response of the PFPeA and PFPrA for all pulse sequences.

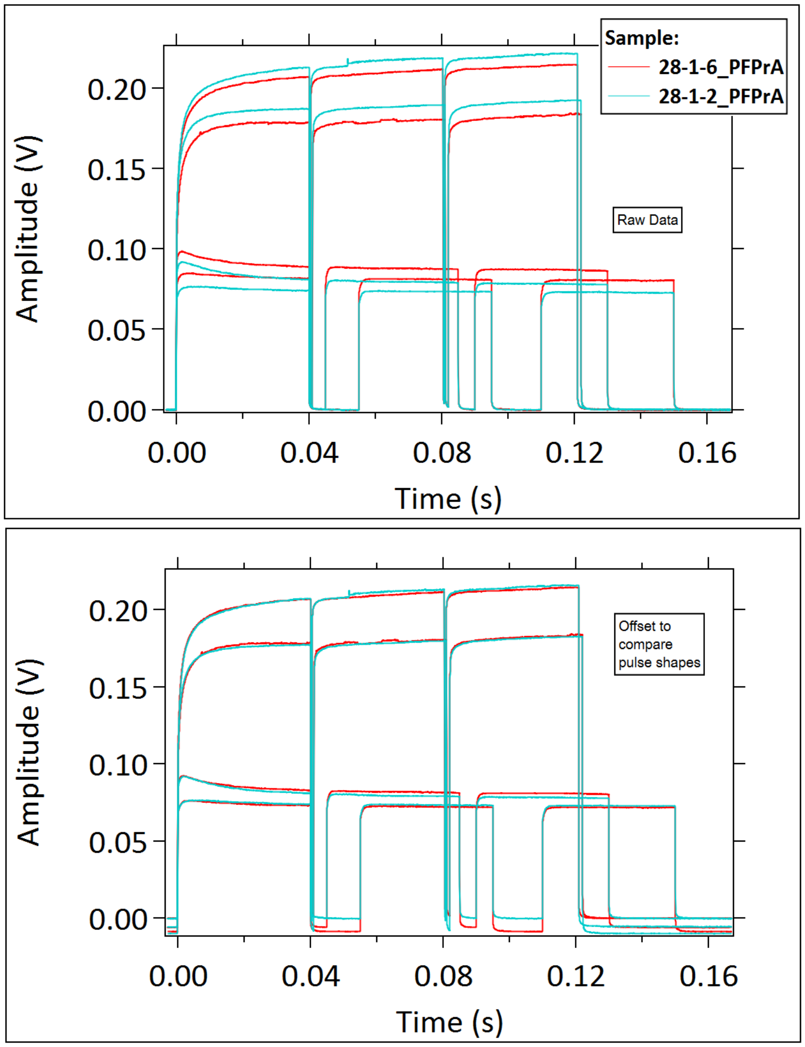

Figure S3b. Pulse response of two different PFPrA sample tests. (Top) raw data; (bottom) Sample 28-1-6 shifted so that the peaks align, showing the similarity in rise times and persistence.

S3. Model Setup and Training

Each complete measurement was composed of four separate CSV files (one file for each pulse sequence of a measurement) with filenames containing device ID, chemical type, and pulse set number corresponding to one of the four led pulse sets. The Python script loaded all CSVs into dictionaries grouping the four pulse set measurements together with the device ID and chemical type as the access key. Once all measurements were loaded, any DC offset within each measurement was removed by averaging the first 100 data points, the period before LED illumination, and subtracting the average point by point from the entire pulse set measurement. The four grouped pulse sequences of each complete measurement were then labeled according to the true class, meaning that any methanol measurements received methanol as their true label that the model prediction would be compared against and so on.

Statistics such as mean, standard deviation, maximum, and minimum were calculated for each region within a measurement. These statistics were taken from a pooled array of channel 1 (the measured ENVIR-OGT current) and channel 2 (the LED driving waveform) values for each region. Through this process, the statistics capture coarse information about the ENVIR-OGT response compared to the LED baseline. The feature extraction process was performed for each region within all four pulse sets of a measurement. For each measurement, features were named by corresponding pulse set, region type, region number, and fit parameter (e.g. "ps1_rise1_a") and placed into rows corresponding to their sample ID.

Python scripts for the one OvR/single, and RF model for both TOF and split out PFAS are located here: **https://github.com/Lukaspy/ENVIR-OGT-Classifiers**

S3.1 Train-test split. The complete pre-processed dataset was divided into training and test groups using an 80/20 stratified shuffle split. Stratification ensured that the class proportions were preserved in both groups, which is important for this relatively small dataset because a purely random split could place many samples of certain chemicals (such as methanol) into the test set, leaving too few in the training set. A fixed random seed was used so that the shuffle and resulting split were reproducible. Sample counts for each class in the train and test sets are provided in Table 1.

S3.2 One versus rest classifier setup. Chemical detection was performed using a one versus rest (OvR) approach. In this method, an independent binary classifier is trained for each chemical to determine whether that chemical is present or absent in a sample. Each model outputs one of three labels: present, absent, or inconclusive. This structure allows each chemical to be evaluated independently. If a sample does not contain any of the trained chemicals, none of the OvR models force a label.

S3.3 Random Forest training and feature selection. Each OvR classifier was implemented using the Random Forest (RF) classifier from Python's scikit-learn library (This page has the citation for a paper describing the scikit-learn library we use heavily for the random forests and other ML tasks: https://scikit-learn.org/stable/about.html.). For each chemical, an initial RF model was trained using all engineered features, and feature importances from this model were used to rank features. The top 200 most important features were retained, and a final RF model was trained using only these features.

Pandas library (used for organizing the data within the script): https://pandas.pydata.org/about/citing.html

SciPy library (used for curve fitting): https://scipy.org/citing-scipy/

matplotlib (used for confusion matrices): https://matplotlib.org/1.4.3/citing.html

S3.4 Threshold determination and cross-validation. To generate present, absent, and inconclusive labels, a detection threshold was learned independently for each OvR classifier using only the training data. Thresholds were optimized using cross-validation (CV) within the 80 percent training group. The training data was split seven times, each with a different shuffle, into a 70 percent fit subgroup and a 30 percent validation subgroup. For each split, a temporary RF model was trained on the fit subgroup and used to produce prediction probabilities for the validation subgroup.

For every CV split, a precision-recall (P-R) curve was computed to evaluate the tradeoff between precision (accuracy of positive predictions) and recall (ability to detect true positives). The threshold corresponding to the maximum F1 score on the P-R curve was selected. The F1 score measures the balance between precision and recall, with values closer to one indicating better performance. This procedure selects the threshold that best balances false positives and false negatives for that split. The final threshold for each chemical was obtained by taking the median of the seven CV-derived thresholds, which helps reduce overfitting. Predicted probabilities within ±0.03 of the final threshold were labeled as inconclusive.

S3.5 Evaluation on test data. After all OvR models were trained, each sample in the held-out test set was evaluated by each binary classifier. Output probabilities, distances from the decision threshold, and final labels were saved to a "lab report" CSV file for downstream analysis. Additional metrics, including learned thresholds, false negative rates, precision, recall, and F1 scores for each class, were saved to summary CSV files.

S3.6 Repeated trials for stability assessment. The entire training and evaluation process was repeated for five independent trials, each using a different random shuffle before the train-test split. These repetitions were used to assess model stability and generalizability with respect to the specific samples selected for training.

S3.7 Combined PFAS Model. A separate multi-class RF classifier was trained for comparison with the OvR scheme. This model assigned each sample to the class with the highest probability (argmax rule), resulting in a forced single-label prediction. All preprocessing and feature extraction steps were identical to the OvR approach. Although this method cannot identify mixtures or samples lacking any learned analyte, it provides insight into the inherent separability of the classes and highlights feature overlap through the resulting confusion matrix. Five trials with different random shuffles were performed to assess model sensitivity and generalizability.

In addition to the OvR and simplified classifier described above, a total organic fluorine (TOF) model was developed to evaluate performance for detection of combined TOF, rather than individual chemicals. Through this, the model is trained to learn the features that fluorinated samples share in common instead of focusing on differentiation of structurally similar PFAS. A TOF OvR model was trained in addition to a TOF classifier using the single random forest classifier setup using the same preprocessing, feature selection, and validation procedures described in the preceding sections. The only modification was that during data labeling all PFAS samples were merged into a single group rather than being labeled individually with the other three alcohol classes remaining unchanged.

S3.8 Accuracy Calculation. Accuracy is defined as:

Accuracy = (TP+TN)/(P+N)

Definitions:

TP = true positive; count of samples where actual sample is positive and it is correctly predicted as positive.

TN = true negative; count of samples where actual sample is negative and it is correctly predicted as negative.

FP = false positive; actual sample is negative but incorrectly predicted as positive.

FN = false negative; actual sample is positive but incorrectly predicted as negative.

P = number of positive samples

N = number of negative samples

The accuracy can also be represented by:

Accuracy = (TPR · P + TNR · N)/(P + N)

where:

TPR = True Positive Rate = TP/(TP + FN)

TNR = True Negative Rate = TN/(TN+FP)

FPR = False Positive Rate = 1 – TNR

FNR = False Negative Rate = 1 – TPR

(F1 = 2 × (precision × recall) / (precision + recall)).

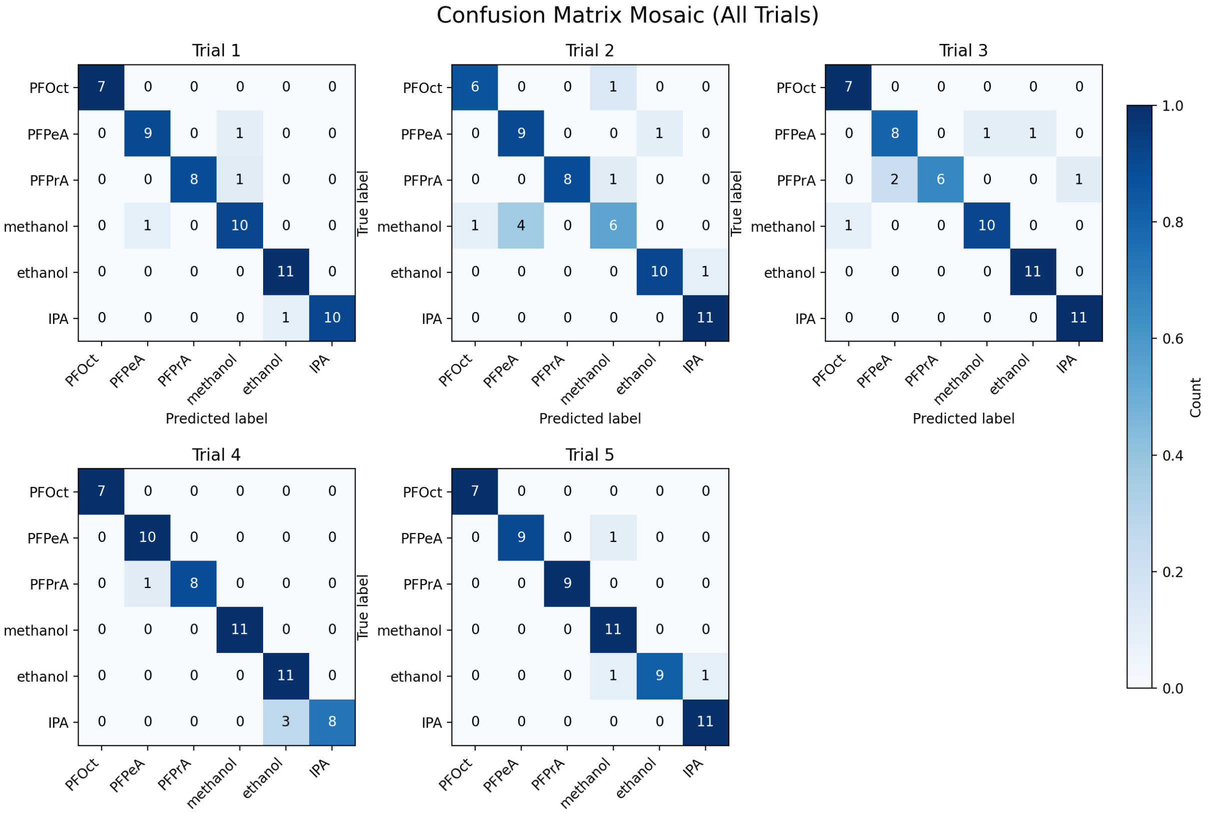

Figure S4. Confusion matrices for five independent trials.

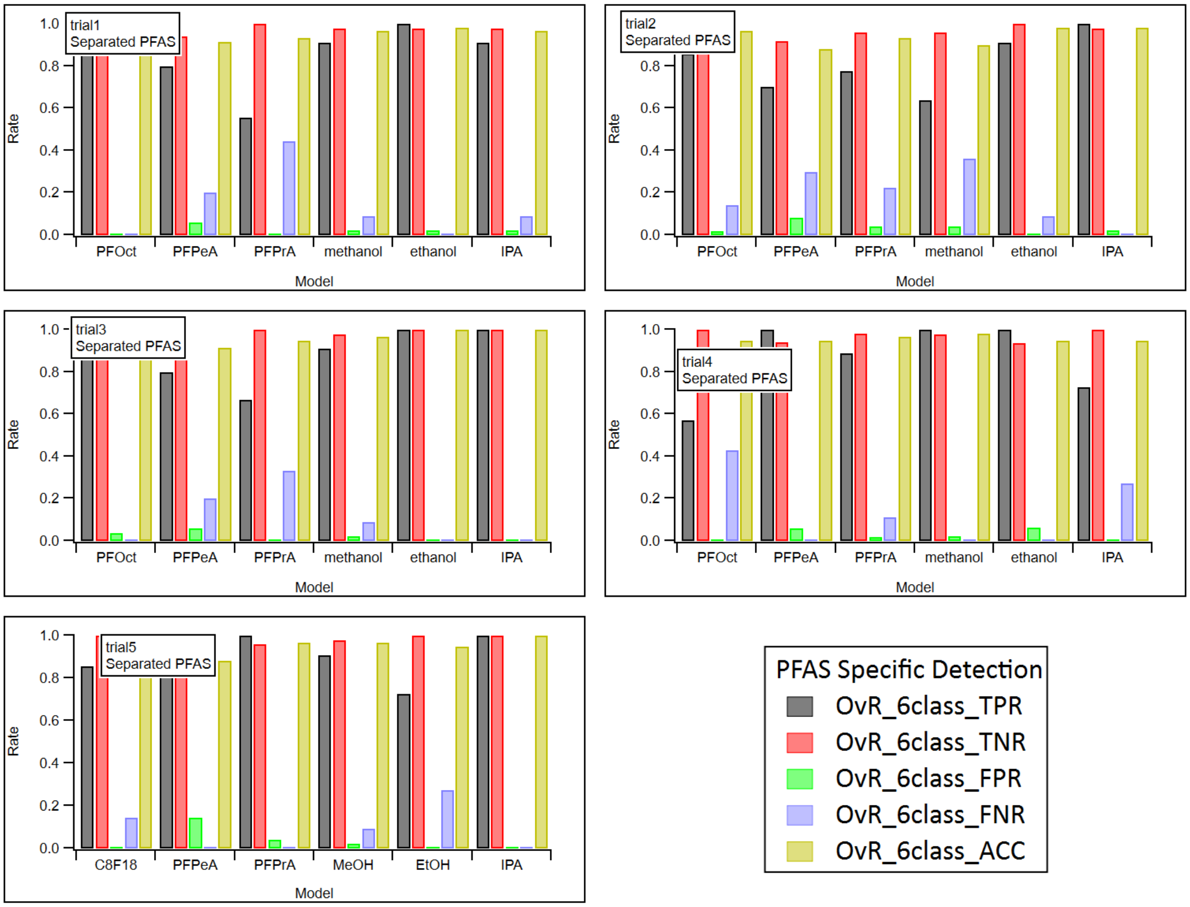

Figure S5. Detection rate performance for five independent trials for all PFAS type detection.

Table S1. Performance rate values for combined five trial data for individual PFAS detection.

Chemical	TPR_mean	TPR_sdev	FNR_mean	FNR_sdev	FPR_mean	FPR_sdev	TNR_mean	TNR_sdev	ACC_mean	ACC_sdev	PREC_mea	PREC_sdev	REC_mean	REC_sdev	F1_mean	F1_sdev
PFOct	0.9714	0.0639	0.0286	0.0639	0.0077	0.0105	0.9923	0.0105	0.9898	0.0152	0.9464	0.0736	0.9714	0.0639	0.9581	0.0634
PFPeA	0.9000	0.0707	0.1000	0.0707	0.0327	0.0310	0.9673	0.0310	0.9559	0.0308	0.8603	0.1176	0.9000	0.0707	0.8765	0.0806
PFPrA	0.8667	0.1217	0.1333	0.1217	0.0000	0.0000	1.0000	0.0000	0.9797	0.0186	1.0000	0.0000	0.8667	0.1217	0.9247	0.0742
ethanol	0.9455	0.0813	0.0545	0.0813	0.0250	0.0228	0.9750	0.0228	0.9695	0.0142	0.9056	0.0767	0.9455	0.0813	0.9204	0.0346
methanol	0.8727	0.1885	0.1273	0.1885	0.0292	0.0186	0.9708	0.0186	0.9525	0.0439	0.8677	0.0932	0.8727	0.1885	0.8654	0.1391
IPA	0.9273	0.1185	0.0727	0.1185	0.0125	0.0114	0.9875	0.0114	0.9763	0.0152	0.9500	0.0456	0.9273	0.1185	0.9328	0.0507

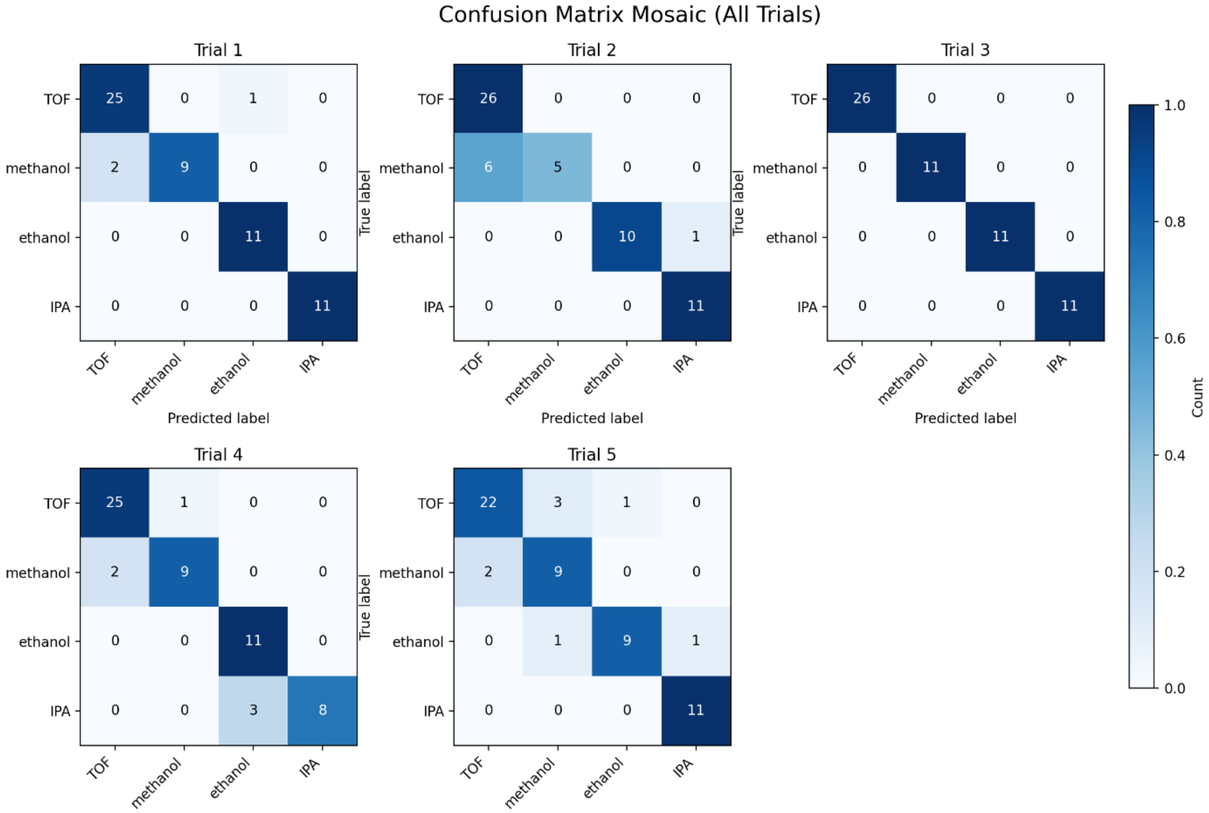

Figure S6. Confusion matrices for five independent trials looking for TOF and alcohols.

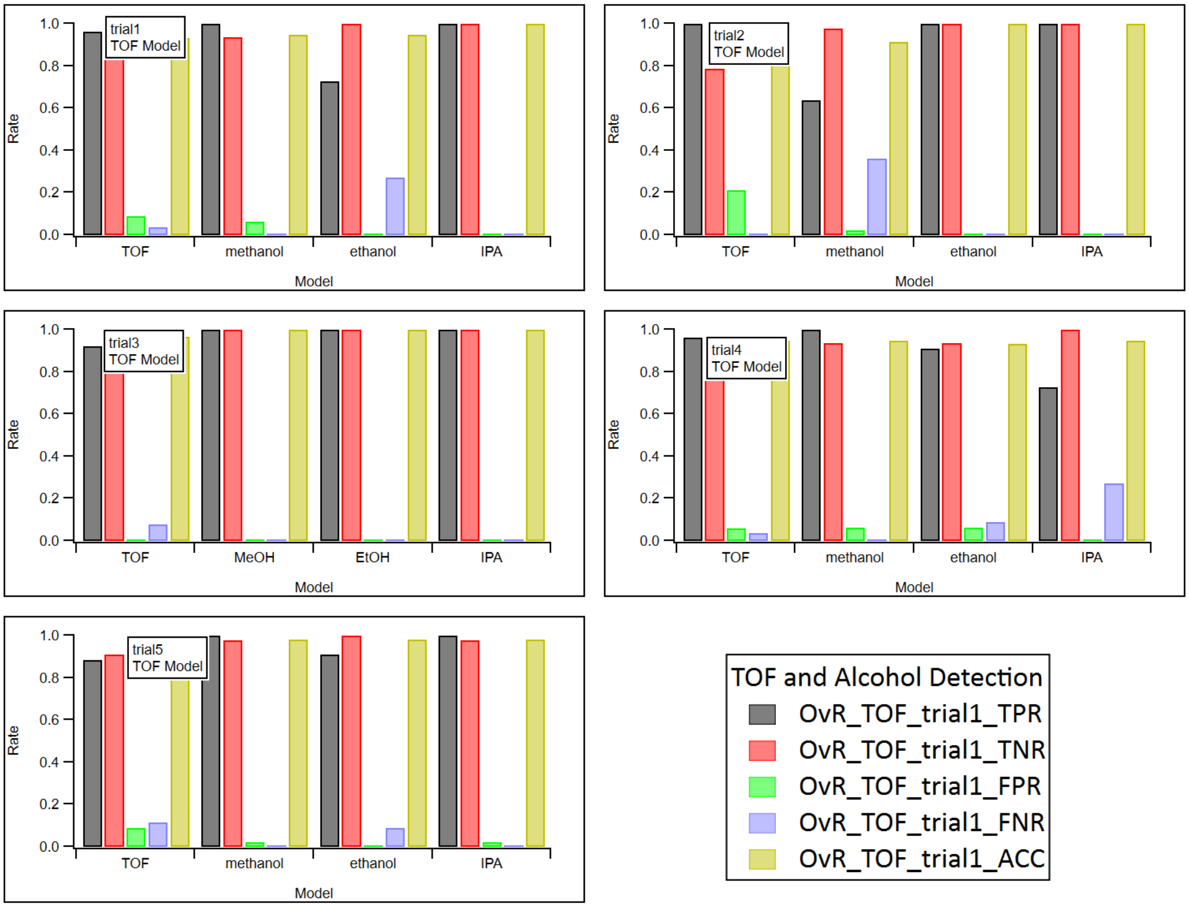

Figure S7. Detection rate performance for five independent trials looking for TOF and alcohols.

Table S2. Performance rate values for combined five trial data for total detection.

Chemical	TPR_mean	TPR_sdev	FNR_mean	FNR_sdev	FPR_mean	FPR_sdev	TNR_mean	TNR_sdev	ACC_mean	ACC_sdev	PREC_mea	PREC_sdev	REC_mean	REC_sdev	F1_mean	F1_sdev
TOF	0.9538	0.0632	0.0462	0.0632	0.0727	0.0664	0.9273	0.0664	0.9390	0.0425	0.9162	0.0670	0.9538	0.0632	0.9327	0.0470
ethanol	0.9455	0.0813	0.0545	0.0813	0.0208	0.0255	0.9792	0.0255	0.9729	0.0227	0.9205	0.0884	0.9455	0.0813	0.9292	0.0590
methanol	0.7818	0.1992	0.2182	0.1992	0.0208	0.0361	0.9792	0.0361	0.9424	0.0442	0.9185	0.1336	0.7818	0.1992	0.8264	0.1440
IPA	0.9455	0.1220	0.0545	0.1220	0.0083	0.0114	0.9917	0.0114	0.9831	0.0208	0.9667	0.0456	0.9455	0.1220	0.9510	0.0647
